# Risk of hip fracture in meat-eaters, pescatarians, and vegetarians: a prospective cohort study of 413,914 UK Biobank participants

**DOI:** 10.1186/s12916-023-02993-6

**Published:** 2023-07-27

**Authors:** James Webster, Darren C. Greenwood, Janet E. Cade

**Affiliations:** 1 Nutritional Epidemiology Group, School of Food Science and Nutrition, University of Leeds, Leeds, LS2 9JT UK; 2 School of Medicine, University of Leeds, Leeds, LS2 9JT UK

**Keywords:** Vegetarian, Plant-based, Cohort, Fracture, Dietary patterns, Diet, Nutrition, Bone, Osteoporosis

## Abstract

**Background:**

Meat-free diets may be associated with a higher risk of hip fracture, but prospective evidence is limited. We aimed to investigate the risk of hip fracture in occasional meat-eaters, pescatarians, and vegetarians compared to regular meat-eaters in the UK Biobank, and to explore the role of potential mediators of any observed risk differences.

**Methods:**

Middle-aged UK adults were classified as regular meat-eaters (*n* = 258,765), occasional meat-eaters (*n* = 137,954), pescatarians (*n* = 9557), or vegetarians (*n* = 7638) based on dietary and lifestyle information at recruitment (2006–2010). Incident hip fractures were identified by record linkage to Hospital Episode Statistics up to September 2021. Multivariable Cox regression models were used to estimate associations between each diet group and hip fracture risk, with regular meat-eaters as the reference group, over a median follow-up time of 12.5 years.

**Results:**

Among 413,914 women, 3503 hip fractures were observed. After adjustment for confounders, vegetarians (HR (95% CI): 1.50 (1.18, 1.91)) but not occasional meat-eaters (0.99 (0.93, 1.07)) or pescatarians (1.08 (0.86, 1.35)) had a greater risk of hip fracture than regular meat-eaters. This is equivalent to an adjusted absolute risk difference of 3.2 (1.2, 5.8) more hip fractures per 1000 people over 10 years in vegetarians. There was limited evidence of effect modification by BMI on hip fracture risk across diet groups (*p*_interaction_ = 0.08), and no clear evidence of effect modification by age or sex (p_interaction_ = 0.9 and 0.3, respectively). Mediation analyses suggest that BMI explained 28% of the observed risk difference between vegetarians and regular meat-eaters (95% CI: 1.1%, 69.8%).

**Discussion:**

Vegetarian men and women had a higher risk of hip fracture than regular meat-eaters, and this was partly explained by their lower BMI. Ensuring adequate nutrient intake and weight management are therefore particularly important in vegetarians in the context of hip fracture prevention.

**Trial registration:**

NCT05554549, registered retrospectively.

**Supplementary Information:**

The online version contains supplementary material available at 10.1186/s12916-023-02993-6.

## Background

Global population growth and longevity increase the number of older adults worldwide. Prevalence of chronic diseases, including frailty, osteoporosis, and sarcopenia, is therefore rising, which increases the risk of falls and fractures [1]. Hip fractures result in a significant loss of independence and quality of life, risk of refracture, other chronic illnesses, and premature mortality. Long hospitalisation periods after a hip fracture also accrue a significant economic burden to healthcare systems (£2–3 billion and $6 billion annually in the UK and US, respectively) [2]. Reducing the risk of hip fracture is therefore a public health priority.

Meat-free diets are becoming more popular in developed countries due to perceived health benefits as well as environmental and ethical concerns [3]. Evidence suggests that vegetarians may have a lower risk of some chronic diseases compared to meat-eaters, including cancer and cardiovascular disease, but a higher risk of fractures [4–6]. However, data from large prospective studies on risk of hip fracture in vegetarians are limited, because few cohorts have recruited sufficient numbers of vegetarians [7]. Two previously published cohort studies in the UK that included mostly women found a greater risk of hip fracture in vegetarians compared to meat-eaters [6, 8]. In contrast, an American cohort of 7th-day Adventists found no clear difference in risk of hip fracture between vegetarians and meat-eaters, but identified cases by self-reported questionnaires [9]. Similarly, one recent study in 126,000 UK Biobank participants reported no difference in hip fracture risk between the highest and lowest quartiles of adherence to a healthful plant-based diet (PBD), where meat and fish intake were considered unhealthy [10]. However, even participants in the highest quartile of adherence to the healthful PBD index ate meat 5.6 times per week, on average. More prospective evidence is required to understand if vegetarian diets, where meat and fish intake are avoided entirely, are associated with hip fracture risk, and more evidence is needed in men, for whom data is scarce.

Risk differences between vegetarians and meat-eaters are plausible due to differences in dietary, anthropometric, and hormonal factors, but remain underexplored. Previous studies report lower intakes of nutrients related to musculoskeletal health, including protein, vitamin D, and vitamin B12 [8, 11, 12]. Studies also report lower body mass index (BMI) and poorer musculoskeletal outcomes in vegetarians, including bone mineral density (BMD), fat-free mass (FFM), and muscle strength [13, 14], which each increase hip fracture risk [15–17]. Additionally, observational studies have shown lower insulin-like growth factor-1 (IGF-1) levels in vegetarians than in meat-eaters [18], potentially due to lower protein intakes [19]; IGF-1 has been positively associated with BMD and inversely associated with hip fracture risk [20]. No study has assessed the role of these factors in explaining any risk differences between diet groups, which could help inform strategies for mitigating any observed risk differences.

We therefore aimed to investigate the risk of hip fracture in occasional meat-eaters, pescatarians, and vegetarians compared to regular meat-eaters in the UK Biobank. We also aimed to determine the roles of BMI, FFM, heel BMD, hand grip strength, IGF-1, and serum vitamin D levels as potential mediators of any observed risk differences.

## Methods

We followed the Strengthening the Reporting of Observational Studies in Epidemiology – Nutritional Epidemiology (STROBE-nut) guidelines for the reporting of cohort studies (Additional file 1: Table S1) [4, 7, 8, 21–31].

### Study design and participants

The UK Biobank is a large prospective cohort of over 500,000 adults across England, Scotland, and Wales, aged 40–69 years at recruitment in 2006–2010. Participants were recruited via National Health Service (NHS) patient registers and attended one of 22 assessment centres across the UK, where participants completed a touchscreen questionnaire, verbal interview, physical measures, and a biosample collection. A full description of the UK Biobank study rationale and design is available elsewhere [32]. Ethical approval was granted from the NHS North West Multicentre Research Ethics Committee (21/NW/0157), and participants provided informed consent for data linkage to health records.

Participants were excluded from this analysis if they had a previous hip fracture (*n* = 1263) or osteoporosis (*n* = 2826) on or before the date of recruitment, were lost to follow-up (*n* = 1260), their genetic sex did not match their reported sex (*n* = 372), their BMI was implausible (< 10 or ≥ 60 kg/m^2^, *n* = 3161), or they were unable to be classified into a diet group due to insufficient data on meat and fish intake (*n* = 4257). This left a total of 489,703 participants potentially eligible for inclusion in this study (Additional file 1: Fig S1).

### Diet group

At recruitment, participants completed a touchscreen food frequency questionnaire (FFQ) that asked about their frequency of consumption of various meat, fish, eggs, and dairy products. Participants were invited to attend an assessment centre for a repeat visit to complete the same questionnaire again in 2012–2013, 2014, and 2019. Similarly to our previous study on this topic in the UK Women’s Cohort Study (UKWCS) [8], participants were then classified as regular meat-eaters (ate meat ≥ 5 times/week), occasional meat-eaters (ate meat < 5 times/week), pescatarians (ate fish but not meat), vegetarians (ate eggs or dairy but not meat or fish), or vegans (did not eat meat, fish, eggs, or dairy) at recruitment and at the latest point of available follow-up for each participant. Vegans were combined with the vegetarian group due to the small number of vegan participants (10 cases/400 participants). Diet group classifications at recruitment were used to represent participants’ diet group during follow-up. Further details on the questionnaire, diet group classification, and agreement of diet group at recruitment and follow-up are provided in Additional file 1: Supplementary methods and Table S2.

### Outcome ascertainment

First incidence of hip fracture was identified using hospital inpatient data for England, Scotland, and Wales (International Classification of Diseases, ICD-9 code 820, and ICD-10 codes S72.0 – S72.2). This included Hospital Episode Statistics for England from 1997 until September 2021, Scottish Morbidity Records for Scotland from 1981 until July 2021, and the Patient Episode Database for Wales from 1998 until February 2018. The timeframe was person-years until hip fracture incidence, or until end of study period or death in those without a hip fracture, calculated as age at time of event or censoring minus age at study entry [33].

### Statistical analyses

#### Main analyses

All statistical methods were pre-registered on ClinicalTrials.gov (NCT05554549).

Dietary, lifestyle, socio-demographic, anthropometric, and other relevant characteristics of UK Biobank participants at recruitment were summarised across diet groups for all participants, and separately for men and women. Cox proportional hazard regression models were used to estimate hazard ratios (HR) and 95% confidence intervals (95% CI) for potential associations between diet groups and hip fracture risk, with regular meat-eaters as the reference group. The target estimand was the relative causal effect of each diet group on hip fracture risk compared with regular meat-eaters.

Unadjusted and multivariable-adjusted models were applied, with attained age as the timescale [33]. Additional confounders included in the adjusted model were determined from a directed acyclic graph (DAG) and included (all at recruitment) the following: region (England, Scotland, Wales), sex (male, female), ethnicity (white, black, Asian, mixed, other), Townsend Deprivation Index (continuous), live alone (yes, no), smoking status (current, former, never), any regular nutritional supplementation (yes, no), total metabolic equivalent task (MET)-minutes of physical activity per week (continuous), alcohol consumption in drinks per day (continuous), BMI (continuous), and history of diabetes (yes, no), cancer (yes, no), cardiovascular disease (CVD; yes, no), or fractures at sites other than the hip (yes, no). Female-specific confounders included the following: number of children (0, 1, 2, 3, ≥ 4 children), menopausal status (premenopausal, postmenopausal), and hormone replacement therapy (HRT) use (current, former, never). The DAG and further information on the classification of covariates are available in Additional file 1: Supplementary Methods. The proportional hazards assumption was checked graphically using the Schoenfeld residuals and log(-log) survival plot methods, and no violations were observed (Additional file 1: Figs S3 and S4).

To estimate the population impact of each diet group on hip fracture risk, absolute risk differences were generated between each diet group and regular meat-eaters (reference group). Predicted incidences for each diet group were calculated using HRs and 95% CIs expressed as floating absolute risks [7, 25, 26]. Absolute risk differences between each diet group and regular meat-eaters were then calculated as the crude difference between the predicted incidence in each diet group versus regular meat-eaters, and were expressed per 1000 people over 10 years. Further details of this method are described in Additional file 1: Supplementary Methods and elsewhere [7].

#### Subgroup analyses

To determine the roles of age (continuous, and dichotomised at < 60, ≥ 60 years), sex (male, female), and BMI (continuous, and dichotomised at ≤ 22.5, > 22.5 kg/m^2^) as potential effect modifiers, we used likelihood ratio tests comparing adjusted Cox regression models with and without an interaction term between diet groups and each subgroup variable. In each case, the potential effect modifier was omitted from the adjustment set.

### Mediation analyses

We explored the potential of selected anthropometric (BMI, heel BMD, FFM, and hand grip strength) and biomarker measures (serum vitamin D and IGF-1) (all continuous variables measured at recruitment) as effect mediators of any significant association(s) between diet group and hip fracture risk. These variables have each been associated with diet groups and hip fracture risk previously [13, 15, 17, 18, 20, 34–36]. Multiple linear regression models, adjusted for relevant confounders (Additional file 1: Supplementary Methods), were applied to compare each potential mediator across diet groups.

The inverse odds-ratio weighting (IORW) method was used to test for causal mediation, which aims to decompose diet group—hip fracture associations (total effect, TE) into estimated associations that are mediated by the potential mediator of interest (natural indirect effect, NIE), or are not mediated by the potential mediator of interest (natural direct effect, NDI) [28]. The proportion of any diet group-hip fracture association mediated by a given anthropometric or biomarker variable of interest (% mediation) was calculated as the natural log of the HR^NIE^ divided by the natural log of the HR^TE^. We did not test for mediation if there was no significant difference in hip fracture risk for a given diet group compared to regular meat-eaters, or if there was no significant difference between diet groups in the anthropometric or biomarker mediator of interest. All mediation analyses are described in detail in Additional file 1: Table S3 and Supplementary Methods.

#### Sensitivity analyses

To determine if any association in vegetarians could be affected by vegans in that group, we fitted an adjusted model with vegetarians and vegans separated. Additional sensitivity analyses were as follows: excluding participants with a survival time < 3 years to check for reverse causation; excluding participants on long-term treatment for illness who may be generally less healthy than the UK population; adjusting for height and weight together rather than BMI; and accounting for death during follow-up as a potentially competing risk. Participants with missing data for a variable required in a given analysis were excluded from that analysis. We also repeated the primary analysis using multiple imputation via chained equations for missing covariate data using 100 imputations under the assumption of missing at random, and combined analytical results using Rubin’s Rule. All statistical analyses were performed using Stata (version 17).

## Results

### Participants

Of 489,703 participants potentially eligible at recruitment, those with missing covariate data for ethnicity (*n* = 2183), SES (*n* = 600), live alone (*n* = 3775), smoking status (*n* = 1737), supplement use (*n* = 1391), physical activity (*n* = 56,753), number of children (*n* = 248), menopausal status (*n* = 1830), and HRT use (*n* = 15,052) were excluded, leaving 413,914 participants for unadjusted and adjusted analyses. The study flow chart is given in Additional file 1: Fig S1.

### Descriptive data

Characteristics of the 413,914 UK Biobank participants at recruitment stratified by diet group are summarised in Table 1. Over a median follow-up time of 12.5 years, 3503 hip fractures were observed (5,034,336 person-years total), corresponding to 0.8% of the cohort. On average, at recruitment, pescatarians and vegetarians were younger than meat-eaters, though time to hip fracture and age at hip fracture were similar across diet groups (Additional file 1: Fig S5). Pescatarians and vegetarians reported higher education levels and were more likely to report living alone (Table 1). The proportion of vegetarians of Asian ethnicity (1184 (15.5%)) was higher than that of regular meat-eaters (3970 (1.5%)). BMI was lower in pescatarians and vegetarians (25.6 (4.6)) kg/m^2^ than in regular meat-eaters (27.8 (4.8) kg/m^2^). Physical activity levels were similar across diet groups. History of diabetes, CVD, and cancer at recruitment was lower in vegetarians than in regular meat-eaters, and there was no difference in history of other fractures at recruitment across diet groups. Additional file 1: Tables S5 shows characteristics of participants at recruitment across diet groups stratified by sex; both male and female pescatarians and vegetarians had lower BMIs and were younger than regular meat-eaters at recruitment. Dietary characteristics of participants at recruitment, as well as characteristics when including or restricting to participants with missing covariate data, are presented in Additional file 1: Tables S6 and S7 and are summarised in Additional file 1: Supplementary results.

**Table 1:** Characteristics of regular meat-eaters, occasional meat-eaters, pescatarians, and vegetarians in the UK Biobank at recruitment

Characteristics, mean (SD), or *n* (%)	Total	Diet group			
		**Regular meat-eater**	**Occasional meat-eater**	**Pescatarian**	**Vegetarian**
Participants (%)	413,914	258,765 (62.5)	137,954 (33.3)	9557 (2.3)	7638 (1.8)
Cases (%)	3503 (0.8)	2045 (0.8)	1310 (0.9)	78 (0.8)	70 (0.9)
**Socio-demographics**					
Age, years (SD)	56.3 (8.1)	56.1 (8.1)	56.9 (8.0)	53.9 (8.0)	52.9 (7.9)
Sex (%)					
Male	199,688 (48.2)	139,354 (53.9)	54,842 (39.8)	2811 (29.4)	2681 (35.1)
Female	214,226 (51.8)	119,411 (46.1)	83,112 (60.2)	6746 (70.6)	4957 (64.9)
Region (%)					
England	366,964 (88.7)	228,925 (88.5)	122,492 (88.8)	8581 (89.8)	6966 (91.2)
Scotland	29,709 (7.2)	19,130 (7.4)	9616 (7.0)	575 (6.0)	388 (5.1)
Wales	17,241 (4.2)	10,710 (4.1)	5846 (4.2)	401 (4.2)	284 (3.7)
Ethnicity (%)					
White	393,251 (95.0)	247,212 (95.5)	130,780 (94.8)	8977 (93.9)	6282 (82.2)
Black	6113 (1.5)	4109 (1.6)	1824 (1.3)	138 (1.4)	42 (0.5)
Asian	8692 (2.1)	3970 (1.5)	3253 (2.4)	285 (3.0)	1184 (15.5)
Mixed	2402 (0.6)	1445 (0.6)	814 (0.6)	84 (0.9)	59 (0.8)
Other	3456 (0.8)	2029 (0.8)	1283 (0.9)	73 (0.8)	71 (0.9)
Degree-level education (%)	141,274 (47.4)	82,529 (44.6)	49,546 (49.9)	5274 (68.6)	3925 (65.4)
Townsend deprivation index (SD)	− 1.4 (3.0)	− 1.4 (3.0)	− 1.4 (3.0)	− 1.0 (3.1)	− 0.7 (3.1)
Live alone (%)	75,245 (18.2)	41,406 (16.0)	29,930 (21.7)	2287 (23.9)	1622 (21.2)
**Lifestyle**					
Physical activity, MET.mins/week (SD)	2951 (3879)	2984 (3993)	2885 (3689)	3038 (3572)	2895 (3690)
Smoking status (%)					
Current	42,697 (10.3)	28,316 (10.9)	13,188 (9.6)	676 (7.1)	517 (6.8)
Former	143,863 (34.8)	90,750 (35.1)	47,390 (34.4)	3437 (36.0)	2286 (29.9)
Never	227,354 (54.9)	139,699 (54.0)	77,376 (56.1)	5444 (57.0)	4835 (63.3)
Alcohol consumption (drinks/day)	1.2 (1.4)	1.3 (1.5)	1.0 (1.3)	1.0 (1.2)	0.7 (1.2)
Nutritional supplementation (%)	206,442 (49.9)	124,388 (48.1)	72,604 (52.6)	5372 (56.2)	4078 (53.4)
**Anthropometrics**					
BMI, kg/m^2^ (SD)	27.3 (4.7)	27.8 (4.8)	26.7 (4.5)	25.2 (4.2)	25.6 (4.6)
< 18.5 (%)	2070 (0.5)	955 (0.4)	846 (0.6)	149 (1.6)	120 (1.6)
18.5–24.9 (%)	136,230 (32.9)	74,806 (28.9)	52,611 (38.1)	5038 (52.7)	3775 (49.4)
≥ 25 (%)	275,614 (66.6)	183,004 (70.7)	84,497 (61.3)	4370 (45.7)	3743 (49.0)
Height, m (SD)	169.0 (9.3)	169.7 (9.3)	167.8 (9.1)	167.4 (8.7)	167.1 (9.3)
**Comorbidities**					
History of diabetes (%)	36,970 (8.9)	25,162 (9.7)	10,859 (7.9)	395 (4.1)	554 (7.3)
History of cancer (%)	42,641 (10.3)	25,788 (10.0)	15,221 (11.0)	1001 (10.5)	631 (8.3)
History of CVD (%)	46,095 (11.1)	30,129 (11.6)	14,853 (10.8)	609 (6.4)	504 (6.6)
History of other fractures (%)	41,196 (10.0)	25,800 (10.0)	13,560 (9.8)	1026 (10.7)	810 (10.6)
**Female-specific covariates**					
Menopausal status (%)					
Premenopausal	62,162 (29.0)	36,214 (30.3)	21,389 (25.7)	2516 (37.3)	2043 (41.2)
Postmenopausal	152,064 (71.0)	83,197 (69.7)	61,723 (74.3)	4230 (62.7)	2914 (58.8)
HRT use (%)					
Current	13,102 (6.1)	7385 (6.2)	5111 (6.1)	394 (5.8)	212 (4.3)
Former	59,758 (27.9)	33,525 (28.1)	24,129 (29.0)	1331 (19.7)	773 (15.6)
Never	141,366 (66.0)	78,501 (65.7)	53,872 (64.8)	5021 (74.4)	3972 (80.1)
≥ 1 child (%)	172,827 (80.7)	99,652 (83.5)	65,071 (78.3)	4673 (69.3)	3431 (69.2)

Nutritional supplementation refers to regularly consuming any nutritional supplements

*SD* standard deviation, *METs* metabolic equivalents, *BMI* body mass index, *CVD* cardiovascular disease, *HRT* hormone replacement therapy

### Diet groups

**Fig. 1:**
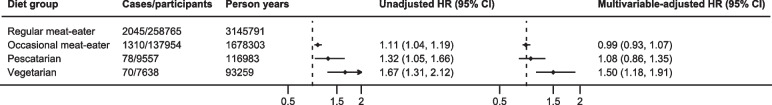
Risk of hip fracture in occasional meat-eaters, pescatarians, and vegetarians compared to regular meat-eaters in the UK Biobank. Both models controlled for age, and the multivariable-adjusted model was adjusted for the following (all at recruitment): region (England, Scotland, Wales), sex (male, female), ethnicity (white, black, Asian, mixed, other), Townsend deprivation index (continuous), live alone (yes, no), smoking (current, former, never), supplementation (yes, no), physical activity in MET-minutes per week (continuous), alcohol consumption in drinks per day (continuous), body mass index (continuous), number of children (0, 1, 2, ≥ 3), menopausal status (premenopausal, postmenopausal), hormone replacement therapy (current, former, never), diabetes (yes, no), cancer (yes, no), cardiovascular disease (yes, no), and other fracture (yes, no). HR (95% CI), hazard ratio (95% confidence interval)

Compared with regular meat-eaters, vegetarians (HR 1.50 (95% CI 1.18, 1.91)) but not occasional meat-eaters (0.99 (0.93, 1.07)) or pescatarians (1.08 (0.86, 1.35)) had a greater risk of hip fracture after adjustment for confounders (Fig. 1), equivalent to 3.2 (1.2, 5.8) more hip fractures in vegetarians for every 1000 people over 10 years (Table 2).

**Table 2:** Adjusted absolute rate differences for hip fracture in occasional meat-eaters, pescatarians, and vegetarians compared to regular meat-eaters in the UK Biobank

Diet group	Predicted incidence per 1000 people over 10 years^a^	Absolute rate difference per 1000 people over 10 years^b^
Regular meat-eater	6.5 (6.2, 6.8)	Reference
Occasional meat-eater	6.5 (6.1, 6.8)	0 (− 0.4, 0.3)
Pescatarian	7.0 (5.6, 8.7)	0.5 (− 0.9, 2.2)
Vegetarian	9.7 (7.7, 12.3)	3.2 (1.2, 5.8)

^a^For regular meat-eaters, calculated as (1 − Sr) × 1000, where Sr = (1 − observed incidence in regular meat-eaters)^10^, representing the predicted 10-year non-incidence or “survival” rate in regular meat-eaters. For other diet groups, calculated as (1 − Sr^HR or 95% CI^) × 1000, where HR or CI are hazard ratios or 95% confidence intervals for hip fracture risk in that diet group, and SR^HR or 95% CI^ represents the predicted 10-year survival rate in each diet group

^b^Calculated as the crude difference between the predicted incidence per 1000 people over 10 years for each diet group and regular meat-eaters

### Subgroup analyses

There was limited evidence of effect modification by BMI on hip fracture risk across diet groups when BMI was modelled categorically (*p*_interaction_ = 0.08), but not when modelled continuously (*p*_interaction_ = 0.5). There was no evidence of effect modification by age (< 60 years vs > 60 years: *p*_interaction_ = 0.9; per 1-year increase: *p*_interaction_ = 0.6) or sex (*p*_interaction_ = 0.9) (Table 3).

**Table 3:** Risk of hip fracture in occasional meat-eaters, pescatarians, and vegetarians compared to regular meat-eaters in UK Biobank participants, stratified by age, sex, and body mass index

Stratifying variable	*n* cases, adjusted HR (95% CI)				*p* interaction
**Age**		** < 60 years**		** ≥ 60 years**	
Regular meat-eaters (reference)	514/152,486	1.00	1531/106,279	1.00	
Occasional meat-eaters	317/75,670	1.03 (0.89, 1.18)	993/62,284	0.98 (0.91, 1.07)	
Pescatarians	31/6747	1.15 (0.80, 1.65)	47/2810	1.04 (0.77, 1.39)	
Vegetarians	32/5770	1.58 (1.10, 2.26)	38/1868	1.45 (1.04, 2.00)	0.9
**Sex**		**Male**		**Female**	
Regular meat-eaters (reference)	883/139,354	1.00	1162/199,411	1.00	
Occasional meat-eaters	381/54,842	0.98 (0.87, 1.10)	929/83,112	1.00 (0.92, 1.09)	
Pescatarians	19/2811	1.29 (0.82, 2.03)	59/6746	1.02 (0.79, 1.33)	
Vegetarians	24/2681	2.04 (1.36, 3.08)	46/4957	1.32 (0.98, 1.78)	0.3
**BMI**		**BMI ≤ 22.5**		**BMI > 22.5**	
Regular meat-eaters (reference)	343/25,794	1.00	1702/232,971	1.00	
Occasional meat-eaters	279/21,297	0.86 (0.74, 1.01)	1031/116,657	1.06 (0.98, 1.15)	
Pescatarians	27/2564	0.90 (0.61, 1.33)	51/6993	1.22 (0.92, 1.61)	
Vegetarians	31/1925	1.61 (1.12, 2.34)	39/5713	1.42 (1.03, 1.96)	0.08

All models controlled for age and were adjusted for the following (all at recruitment): region (England, Scotland, Wales), sex (male, female), ethnicity (white, black, Asian, mixed, other), Townsend deprivation index (continuous), live alone (yes, no), smoking (current, former, never), supplementation (yes, no), physical activity in MET-minutes per week (continuous), alcohol consumption in drinks per day (continuous), body mass index (continuous), number of children (0, 1, 2, ≥ 3), menopausal status (premenopausal, postmenopausal), hormone replacement therapy (current, former, never), diabetes (yes, no), cancer (yes, no), cardiovascular disease (yes, no), and other fracture (yes, no). Each potential effect modifier was omitted from their adjustment set

*HR (95% CI)* hazard ratio (95% confidence interval), *BMI* body mass index

### Mediation analyses

Adjusted and relative means for BMI, heel BMD, FFM, hand grip strength, serum vitamin D, and IGF-1 at recruitment across diet groups are shown in Additional file 1: Table S8. Potential mediation through each of these variables for the observed higher risk of hip fracture in vegetarians compared to regular meat-eaters is shown in Table 4. BMI, FFM, serum vitamin D, and IGF-1 were lower in vegetarians than in regular meat-eaters (Additional file 1: Table S8). BMI was found to partly mediate the observed difference in hip fracture risk between vegetarians and regular meat-eaters, with a decomposed HR^NIE^ of 1.17 (95% CI: 1.00, 1.35), implying that BMI may explain 27.8% (95% CI: 1.1%, 69.8%) of the risk difference (Table 4). There was no clear evidence of mediation through FFM, serum vitamin D, or IGF-1 for the observed risk difference between vegetarians and regular meat-eaters (Table 4). Heel BMD and hand grip strength did not differ significantly between these diet groups (Additional file 1: Table S8) and were not considered in the causal mediation analyses.

**Table 4:** Summary of the total, direct, and indirect effects of potential mediators for differences in hip fracture risk between vegetarians and regular meat-eaters in the UK Biobank

**Vegetarians vs regular meat-eaters**		**Conditional effect, HR or % (95% CI)**			
**Potential mediator**	***n*/*N***	**Total effect**	**Direct effect**	**Indirect effect**	**% mediation**
BMI	2115/266,403	1.77 (1.34, 2.25)	1.51 (1.11, 2.03)	1.17 (1.00, 1.35)	27.8 (1.1, 69.8)
FFM	2056/262,679	1.68 (1.27, 2.13)	1.78 (1.19, 2.44)	0.95 (0.73, 1.21)	− 10.5 (− 77.4, 44.8)
Vitamin D	1874/238,837	1.67 (1.26, 2.10)	1.61 (1.18, 2.13)	1.03 (0.86, 1.23)	6.5 (− 35.4, 45.6)
IGF-1	1949/248,163	1.63 (1.25, 2.06)	1.64 (1.25, 2.08)	1.00 (0.94, 1.06)	− 0.8 (− 16.7, 14.4)

All models controlled for age and were adjusted for the following (all at recruitment): region (England, Scotland, Wales), sex (male, female), ethnicity (white, black, Asian, mixed, other), Townsend deprivation index (continuous), live alone (yes, no), smoking (current, former, never), supplementation (yes, no), physical activity in MET-minutes per week (continuous), alcohol consumption in drinks per day (continuous), number of children (0, 1, 2, ≥ 3), menopausal status (premenopausal, postmenopausal), hormone replacement therapy (current, former, never), diabetes (yes, no), cancer (yes, no), cardiovascular disease (yes, no), and other fracture (yes, no). Models for vitamin D and IGF-1 were also adjusted for BMI, and the model for FFM was adjusted for height

The natural indirect effect represents the estimated association of diet group and hip fracture risk through the potential mediator

The natural direct effect represents the estimated association of diet group and hip fracture risk not through the potential mediator

For each mediator, participants with missing values for that mediator or for relevant covariates were excluded from the analysis

*BMI* body mass index, *FFM* fat-free mass, *IGF-1* insulin-like growth factor-1, *HR (95% CI)* hazard ratio (95% confidence intervals)

### Sensitivity analyses

All sensitivity analyses are presented in Additional file 1: Fig S6 and Table S9. Excluding participants with short follow-up durations (< 3 years) and excluding those on long-term treatment for illness increased the magnitude of the association for vegetarians (1.64 (1.27, 2.11) and 1.91 (1.35, 2.70) respectively) but not for other diet groups, but confidence intervals also widened. Differentiating between vegetarians (60 cases / 7238 participants) and vegans (10 cases / 400 participants) slightly attenuated the estimate for vegetarians (vegetarians: 1.38 (1.06, 1.79); vegans: 3.26 (1.75, 6.08)). For all diet groups, estimates remained similar in the competing risks analysis (Additional file 1: Table S9). Estimates were similar for occasional meat-eaters and vegetarians when missing covariate data were imputed, but the association strengthened for pescatarians (1.29 (1.05, 1.57); Additional file 1: Fig S6).

## Discussion

### Principal findings

In this large prospective British cohort of men and women, there are three important findings: First, vegetarians but not pescatarians or occasional meat-eaters were at a higher risk of hip fracture than regular meat-eaters, but absolute risk differences were modest. These results remained after adjustment for key socio-demographic and lifestyle factors. Second, there was no clear evidence of effect modification by age or sex, and there was limited evidence of effect modification by BMI. Finally, the lower average BMI in vegetarians explained some of the observed risk difference compared to regular meat-eaters, but a large proportion remained unexplained.

### Comparison with previous studies

Only three previously published prospective studies have assessed meat-free diets in relation to hip fracture risk [6, 8, 9]. In the European Prospective Investigation into Cancer-Oxford (EPIC-Oxford) [6], UKWCS [8], and Adventist Health Study-2 (AHS-2) cohorts [9], compared to meat-eaters, vegetarians were at a greater risk in both UK cohorts but not in the AHS-2, whilst pescatarians were at a greater risk in the EPIC-Oxford cohort only. Our findings are consistent with results from the two previous British cohorts on this topic for vegetarians, strengthening the evidence of an increased risk of hip fracture in British vegetarians. In the AHS-2, hip fractures were identified from self-reported questionnaires, which are prone to selective loss to follow-up compared to more deterministic linkage to hospital records used here and in the other UK cohorts, which may contribute to the difference in findings. Importantly, we provide evidence of a greater risk of hip fracture in vegetarian men, which has only been observed in the EPIC-Oxford study in which 77% of vegetarians were women. Similarly to the UKWCS and AHS-2 studies, there was no clear evidence of a risk difference for pescatarians in this study, whereas pescatarians were at a 26% greater risk in the EPIC-Oxford study. These differences may be attributable to differences in fish intake, population characteristics, and other sources of residual confounding across cohorts, although in the sensitivity analysis when we imputed for missing covariate data, the estimate was similar to that observed in the EPIC-Oxford study.

### Interpretation and implications

Whilst the relative increase in hip fracture risk for vegetarians was high (50%), this represents an absolute difference of only 3.2 more cases per 1000 people over 10 years, which is consistent with estimates from the EPIC-Oxford study. This modest absolute risk difference should be weighed against the potential associated health benefits of vegetarian diets for more common conditions when formulating dietary guidelines, including 13 fewer cancers per 1000 people over 10 years and a 9% lower risk of CVD observed previously in the UK Biobank [4, 5]. Evidence of associations for occasional meat-eaters and pescatarians was unclear, but absolute risk differences and their confidence intervals appeared to rule out a clinically relevant benefit or harm.

In this study, vegetarians had a lower BMI (adjusted means of 25.9 vs 27.7 kg/m^2^) and were less likely to be overweight (means of 49.0% vs 70.7%) than regular meat-eaters on average, which is consistent with previous studies [6, 8, 37]. Low BMI is a known risk factor for hip fracture, and overweight (BMI between 25 and 29.9 kg/m^2^) but not obesity (BMI ≥ 30 kg/m^2^) may reduce hip fracture risk [15]. In the subgroup analysis by BMI, the difference in *p*-interaction values when BMI was modelled continuously (*p* = 0.5) compared to when dichotomised at 22.5 kg/m^2^ (p = 0.08) may suggest a potential non-linear interaction of BMI with diet groups on hip fracture risk. However, further investigation of a potentially non-linear interaction of BMI with diet groups was not possible in this study due to the low number of vegetarians and pescatarians with obesity.

In the UKWCS and EPIC-Oxford cohorts [6, 8], adjustment for BMI slightly attenuated risk estimates. We extend these findings by showing through causal mediation analysis that differences in BMI explained approximately 28% of the higher risk in vegetarians. Lower BMI in vegetarians may reflect inadequate fat mass which reduces cushioning from impact forces during a fall. Alternatively, lower BMI may indicate poor musculoskeletal health. Previous studies have reported slightly lower whole-body and femoral neck BMD, FFM, and muscle strength in vegetarians than in meat-eaters [13, 14]. These factors are more common at a lower BMI and increase the risk of hip fracture [15]. Small differences were observed for heel BMD, FFM, and hand grip strength between diet groups in this study, but their roles as potential mediators were unclear. Femoral neck BMD contributes to hip fracture risk more than heel BMD [17], but mediation analysis for femoral neck BMD was not possible since this data was only available in a subset of participants (around 10%). Weight management may therefore help to mitigate some of the increased risk of hip fracture in vegetarians and warrants exploration in future trials. Further studies are needed to understand musculoskeletal health across diet groups and consequences on hip fracture risk. The generally protective role of BMI in hip fracture prevention should also be considered alongside the adverse health effects of overweight [38].

A large proportion of the higher risk of hip fracture in vegetarians was not explained by BMI, implying that other factors are important. Previously published studies have suggested lower circulating vitamin D and IGF-1 levels in vegetarians than in meat-eaters [18, 34], and inverse associations of these biomarkers with hip fracture risk through their effects on bone and muscle health [20, 35]. Circulating vitamin D and IGF-1 were lower in vegetarians than in meat-eaters in this study, but there was no clear evidence of mediation through IGF-1 and vitamin D. Another possible explanation is that vegetarians, on average, have lower intakes of nutrients important to bone and muscle health, such as protein, vitamin D, and vitamin B12 [8, 11, 12]. In this study, vegetarians consumed less dietary protein, iron, iodine, niacin, selenium, vitamin B12, and vitamin D than other diet groups. Specifically, vegetarians were less likely to meet daily recommended protein intakes of 0.75 g/kg body weight/day for adults than regular meat-eaters (68.2% vs 85.2%) [31] and were less likely to achieve higher protein intakes of 1.2 g/kg body weight/day (15.8% vs 33.6%), which may help to attenuate age-related bone and muscle loss [39]. We could not investigate mediation through dietary factors since nutrient data was only available in 50.1% of the cohort. Nevertheless, given that dietary protein has been inversely associated with hip fracture risk in previously published studies [40, 41], and high intakes have been reported to be safe (up to 2 g/kg body weight/day) [41], increasing protein intake may help to reduce hip fracture risk in vegetarians, and warrants exploration in further studies.

### Strengths and limitations

This study has many strengths. The moderately long prospective follow-up and identification of hip fractures by linkage to hospital records minimised outcome misclassification and loss to follow-up. The wide array of lifestyle, hospital, and biomarker data available in the UK Biobank permitted adjustment for many likely confounders and enabled exploration of the roles of anthropometric and biomarker factors as potential mediators of observed associations. In a sub-sample of participants with repeated measurements (*n* = 57,730), there was little evidence of changes in diet groups over time, which minimises the risk of misclassification, and there was little evidence of reverse causality, as results were similar after excluding participants with < 3 years of follow-up. Finally, we provide evidence in men and women.

Our study has important limitations. Vegans (do not eat meat, fish, eggs, or dairy) are less likely to meet nutrient intake recommendations for protein and calcium and may be at a higher risk of hip fractures than meat-eaters [6, 11], but there were not enough vegans in this cohort to assess their risk independently. Further prospective studies into hip fracture risk with a large proportion of vegans are needed. Additionally, diet quality may vary within and between diet groups and may influence hip fracture risk. Future studies should aim to determine if a well-planned vegetarian diet mitigates the observed risk difference. This study focused on the risk of hip fracture across diet groups; further research should investigate if associations vary by fracture site. Participants were, on average, younger at hip fracture or by the end of follow-up than the average age at hip fracture in men (84 years) and women (83 years) [42], which limited the number of cases observed. Moreover, relatively low numbers of older adults could explain why there was no evidence of effect modification by age. We were unable to differentiate between fragility and traumatic hip fractures because data on the cause of hip fractures were not available. However, most hip fractures in middle-aged to older adults are fragility fractures [43], and since risk of traumatic hip fracture is unlikely to differ across diet groups, any outcome misclassification would only dilute risk estimates. As with all observational studies, residual confounding remains possible, and causality cannot be inferred. In mediation analyses, residual confounding is also possible at the exposure-outcome, exposure-mediator, and mediator-outcome levels. Additionally, we used measures of anthropometrics and biomarkers at recruitment for mediators, which may not represent measures during follow-up, though correlations with repeat measures show high agreement. Nevertheless, the mediation results should be interpreted with caution, particularly given the wide confidence intervals for all mediators. UK Biobank participants have a healthy risk profile compared to the British population [44] and are mostly Caucasian. These factors reduce generalisability to the UK population and to other ethnic groups, respectively.

## Conclusion

Vegetarian men and women had a higher risk of hip fracture than regular meat-eaters and were in part explained by their lower BMI, but absolute risk differences were small and should be weighed against the potential health benefits of vegetarian diets. Further work is needed to fully understand mechanisms underpinning risk differences; diet planning and weight management could help to mitigate the risk difference, and warrant exploration in further studies so that policy recommendations can advance.

## Supplementary Information

**Additional file 1:** **Supplementary Figures: **Fig S1: Flow chart of UK Biobank participants for this study. Fig S2: Directed Acyclic Graph showing the relationship between diet group, hip fracture incidence, and related factors. Fig S3: Risk of hip fracture in occasional meat-eaters, pescatarians, and vegetarians compared to regular meat-eaters in the UK Biobank with multiple imputation via chained equations for missing covariate data. Fig S4: Log(-log) survival plot for regular meat-eaters, occasional meat-eaters, pescatarians, and vegetarians in the UK Biobank. Fig S5: A) Time until hip fracture and B) Age at hip fracture in regular meat-eaters, occasional meat-eaters, pescatarians, and vegetarians in the UK Biobank. Fig S6: Risk of hip fracture in occasional meat-eaters, pescatarians, and vegetarians compared to regular meat-eaters in the UK Biobank with multiple imputation via chained equations for missing covariate data. **Supplementary Tables: **Table S1: Strengthening the Reporting of Observational studies in Nutritional Epidemiology (STROBE-Nut) checklist. Table S2: Diet group categorisation and definitions. Table S3: Summary of mediation analyses using the inverse odds ratio weighting method in the UK Biobank. Table S4: Diet group classifications at recruitment and at the latest point of available follow-up in UK Biobank participants. Table S5: Dietary characteristics of UK Biobank participants by diet group at recruitment. Table S6: Characteristics of UK Biobank participants by diet group at recruitment, stratified by sex. Table S7: Characteristics of UK Biobank participants at recruitment that were included or excluded from analyses. Table S8: Adjusted and relative means (95% confidence intervals) of potential mediators at recruitment across diet groups in the UK Biobank. Table S9: Risk of hip fracture by diet group in the UK Biobank with varying restrictions. **Supplementary Methods: **Diet group classification. Other dietary measurements. Derivation of potential mediators. Derivation of covariates. Calculating absolute risk differences. Mediation analyses. **Supplementary results: **Diet group at recruitment and follow-up. Dietary characteristics at recruitment. Descriptive characteristics at recruitment with varying restrictions.

## Data Availability

UK Biobank data are available through application to the database https://www.ukbiobank.ac.uk/.
