# Supplementary material for: Risk of hip fracture in meat-eaters, pescatarians, and vegetarians: a prospective cohort study of 413,914 UK Biobank participants
Source: BMC Med. 2023 Jul 27;21:278. doi: 10.1186/s12916-023-02993-6 (PMC10375740; doi:10.1186/s12916-023-02993-6)
Supplement: Supplementary file 1 — Additional file 1: Supplementary Figures: Fig S1: Flow chart of UK Biobank participants for this study. Fig S2: Directed Acyclic Graph showing the relationship between diet group, hip fracture incidence, and related factors. Fig S3: Risk of hip fracture in occasional meat-eaters, pescatarians, and vegetarians compared to regular meat-eaters in the UK Biobank with multiple imputation via chained equations for missing covariate data. Fig S4: Log(-log) survival plot for regular meat-eaters, occasional meat-eaters, pescatarians, and vegetarians in the UK Biobank. Fig S5: A) Time until hip fracture and B) Age at hip fracture in regular meat-eaters, occasional meat-eaters, pescatarians, and vegetarians in the UK Biobank. Fig S6: Risk of hip fracture in occasional meat-eaters, pescatarians, and vegetarians compared to regular meat-eaters in the UK Biobank with multiple imputation via chained equations for missing covariate data. Supplementary Tables: Table S1: Strengthening the Reporting of Observational studies in Nutritional Epidemiology (STROBE-Nut) checklist. Table S2: Diet group categorisation and definitions. Table S3: Summary of mediation analyses using the inverse odds ratio weighting method in the UK Biobank. Table S4: Diet group classifications at recruitment and at the latest point of available follow-up in UK Biobank participants. Table S5: Dietary characteristics of UK Biobank participants by diet group at recruitment. Table S6: Characteristics of UK Biobank participants by diet group at recruitment, stratified by sex. Table S7: Characteristics of UK Biobank participants at recruitment that were included or excluded from analyses. Table S8: Adjusted and relative means (95% confidence intervals) of potential mediators at recruitment across diet groups in the UK Biobank. Table S9: Risk of hip fracture by diet group in the UK Biobank with varying restrictions. Supplementary Methods: Diet group classification. Other dietary measurements. Derivation of potential me [file 12916_2023_2993_MOESM1_ESM.docx]

**Additional file 1**

Contents

[**Supplementary Figures** 3](#_Toc140665152)

[Fig S1: Flow chart of UK Biobank participants for this study. 3](file:///C:\Users\webst\OneDrive\Documents\Academic\PhD%20Nutritional%20Epidemiology\3.%20UK%20Biobank\5.%20Manuscripts\BMC%20Medicine\Peer%20review%20round%202\Additional%20file%201.docx#_Toc140665153)

[Fig S2: Directed Acyclic Graph showing the relationship between diet group, hip fracture incidence, and related factors. 4](#_Toc140665154)

[Fig S3: Scaled Schoenfeld residuals over analysis time in regular meat-eaters, occasional meat-eaters, pescatarians, and vegetarians in the UK Biobank. 5](#_Toc140665155)

[Fig S4: Log(-log) survival plot for regular meat-eaters, occasional meat-eaters, pescatarians, and vegetarians in the UK Biobank. 6](#_Toc140665156)

[Fig S5: A) Time until hip fracture and B) Age at hip fracture in regular meat-eaters, occasional meat-eaters, pescatarians, and vegetarians in the UK Biobank. 7](#_Toc140665157)

[Fig S6: Risk of hip fracture in occasional meat-eaters, pescatarians, and vegetarians compared to regular meat-eaters in the UK Biobank with multiple imputation via chained equations for missing covariate data. 8](#_Toc140665158)

[**Supplementary Tables** 9](#_Toc140665159)

[Table S1: Strengthening the Reporting of Observational studies in Nutritional Epidemiology (STROBE-Nut) checklist. 9](#_Toc140665160)

[Table S2: Diet group categorisation and definitions. 12](#_Toc140665161)

[Table S3: Summary of mediation analyses using the inverse odds ratio weighting method in the UK Biobank. 13](#_Toc140665162)

[Table S4: Diet group classifications at recruitment and at the latest point of available follow-up in UK Biobank participants. 14](#_Toc140665163)

[Table S5: Dietary characteristics of UK Biobank participants by diet group at recruitment. 15](#_Toc140665164)

[Table S6: Characteristics of UK Biobank participants by diet group at recruitment, stratified by sex. 18](#_Toc140665165)

[Table S7: Characteristics of UK Biobank participants at recruitment that were included or excluded from analyses. 20](#_Toc140665166)

[Table S8: Adjusted and relative means (95% confidence intervals) of potential mediators at recruitment across diet groups in the UK Biobank. 23](#_Toc140665167)

[Table S9: Risk of hip fracture by diet group in the UK Biobank with varying restrictions. 25](#_Toc140665168)

[**Supplementary Methods** 27](#_Toc140665169)

[Diet group classification 27](#_Toc140665170)

[Other dietary measurements 28](#_Toc140665171)

[Derivation of potential mediators 28](#_Toc140665172)

[Derivation of covariates 29](#_Toc140665173)

[Calculating absolute risk differences 33](#_Toc140665174)

[Mediation analyses 33](#_Toc140665175)

[**Supplementary results** 36](#_Toc140665176)

[Diet group at recruitment and follow-up 36](#_Toc140665177)

[Dietary characteristics at recruitment 36](#_Toc140665178)

[Descriptive characteristics at recruitment with varying restrictions 37](#_Toc140665179)

# **Supplementary Figures**

### Fig S1: Flow chart of UK Biobank participants for this study.

**.**

UK Biobank participants recruited between 2006-2010 (n=502,409)

Participants potentially eligible (n=489,703)

- Regular meat-eaters (n=304,576)
- Occasional meat-eaters (n=164,591)
- Pescatarians (11,104)
- Vegetarians (9432)

**Exclusion criteria (n total = 12,706):**

- Unable to be classified into a diet group (4257)
- Had a hip fracture before or on the date of recruitment (n=1263)
- Had osteoporosis before or on the date of recruitment (n=2826)
- Lost to follow-up (n=1260)
- Genetic sex did not match self-reported sex
- Implausible BMI (n=3161) (n=372)

Participants in unadjusted and multivariable-adjusted analyses with complete covariate data (n=413,914)

- Regular meat-eaters (n=258,765)
- Occasional meat-eaters (n=137,954)
- Pescatarians (9557)
- Vegetarians (7638)

**Excluded due to missing data (n total=75,789):**

- Ethnicity (n=2183)
- Townsend Deprivation Index (n=600)
- Live alone (n=3775)
- Smoking status (n=1737)
- Nutritional supplementation (n=1391)
- Physical activity (n=56,753)
- Number of children (n=248)
- Menopausal status (n=1830)
- HRT use (n=15,052)

**Figure S1: Flow chart of UK Biobank participants for this study.**


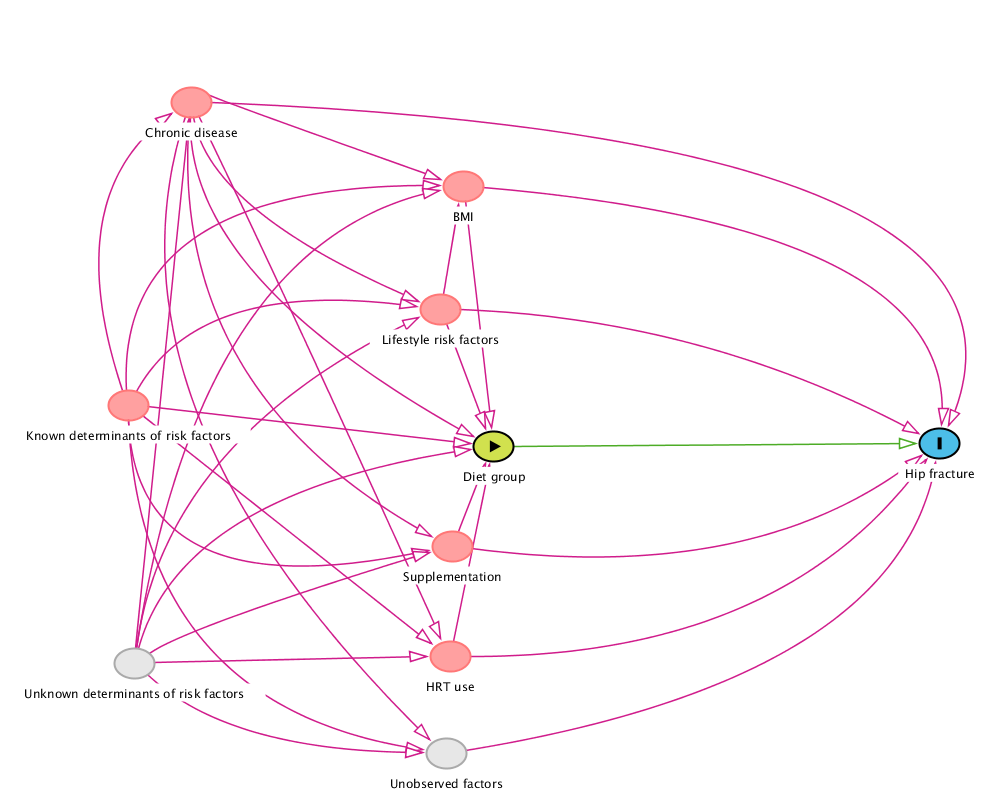


Fig S2: Directed Acyclic Graph showing the relationship between diet group, hip fracture incidence, and related factors. Adapted from Webster et al. (2022) (8). The green oval represents diet group (exposure), and the blue oval represents hip fracture incidence (outcome). Pink nodes are ancestors of the exposure and outcome (confounders), whilst grey nodes are unknown or unmeasured confounders. The green line represents the causal link of interest, whilst pink lines are biasing paths. Known determinants of risk factors include age, region, ethnicity, socio-economic status, living alone/marital status, menopausal status, and number of children. Lifestyle risk factors include physical activity, smoking, and alcohol intake. Supplementation refers to use of any nutritional supplements. CD: chronic disease, defined as prevalence of cardiovascular disease, cancer, diabetes, osteoporosis, and prior fracture (at any site).


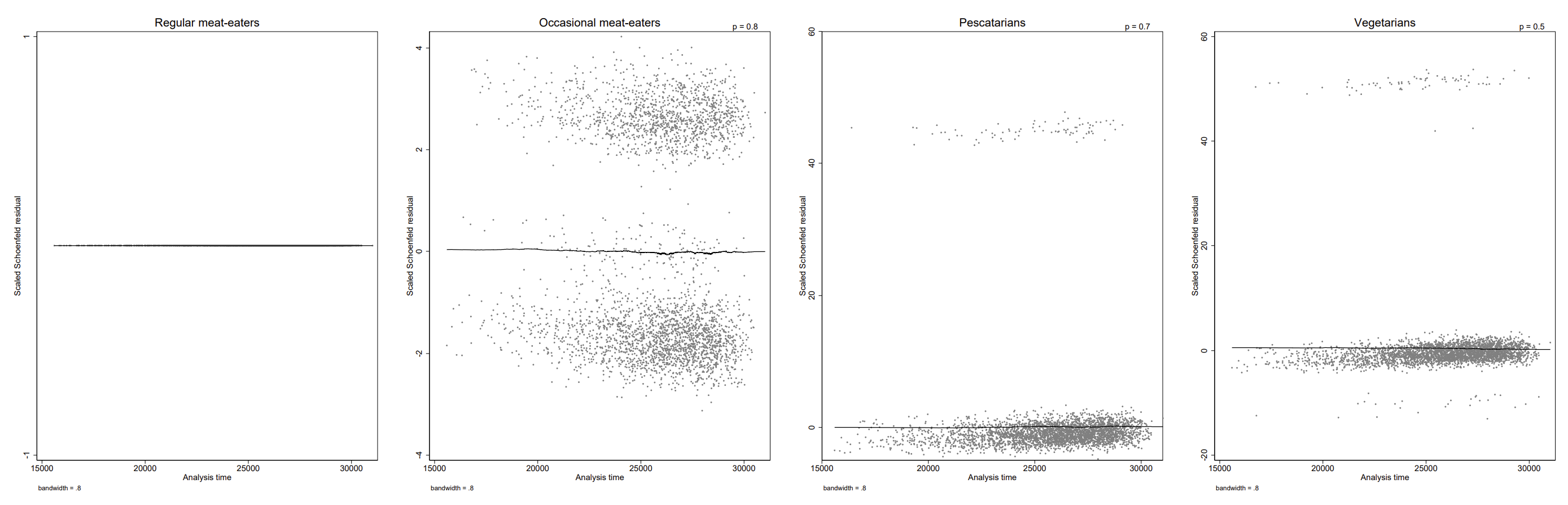


### Fig S3: Scaled Schoenfeld residuals over analysis time in regular meat-eaters, occasional meat-eaters, pescatarians, and vegetarians in the UK Biobank.


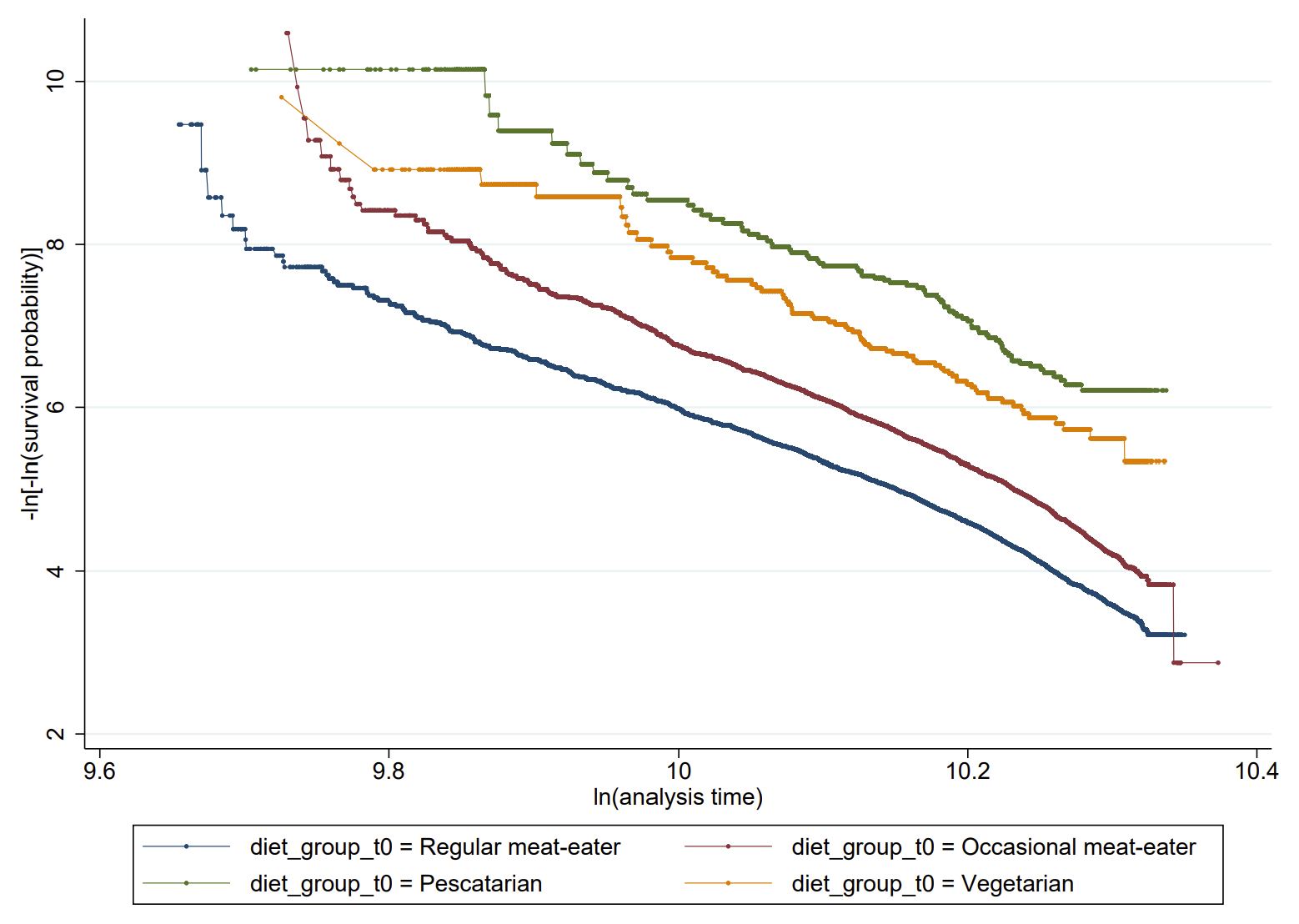


### Fig S4: Log(-log) survival plot for regular meat-eaters, occasional meat-eaters, pescatarians, and vegetarians in the UK Biobank.


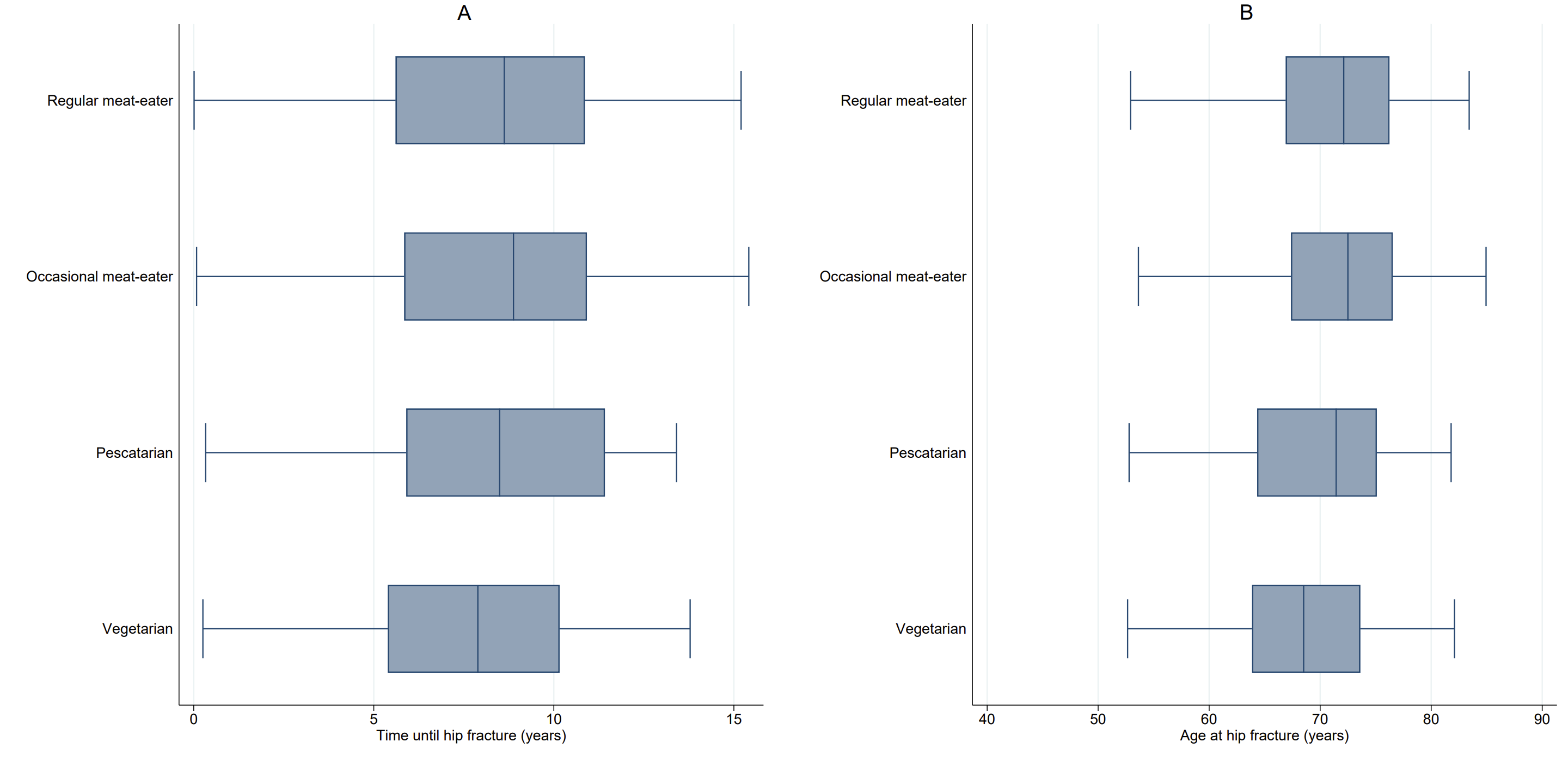


Fig S5: A) Time until hip fracture and B) Age at hip fracture in regular meat-eaters, occasional meat-eaters, pescatarians, and vegetarians in the UK Biobank. The solid line within each box represents the median, and the outer box lines represent the interquartile range.


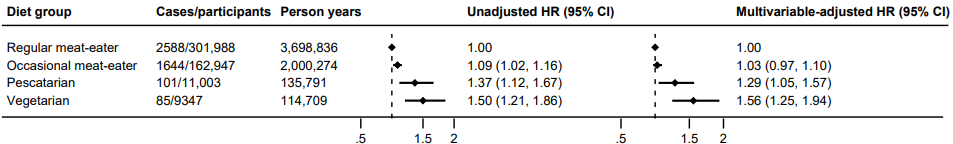


Fig S6: Risk of hip fracture in occasional meat-eaters, pescatarians, and vegetarians compared to regular meat-eaters in the UK Biobank with multiple imputation via chained equations for missing covariate data. Both models controlled for age, and the multivariable-adjusted model was adjusted for the following (all at recruitment): region (England, Scotland, Wales), sex (male, female), ethnicity (white, black, Asian, mixed, other), Townsend deprivation index (continuous), live alone (yes, no), smoking (current, former, never), supplementation (yes, no), physical activity in MET-minutes per week (continuous), alcohol consumption in drinks per day (continuous), body mass index (continuous), number of children (0, 1, 2, ≥ 3), menopausal status (premenopausal, postmenopausal), hormone replacement therapy (current, former, never), diabetes (yes, no), cancer (yes, no), cardiovascular disease (yes, no), and other fracture (yes, no). HR (95% CI): hazard ratio (95% confidence interval).

# **Supplementary Tables**

### Table S1: Strengthening the Reporting of Observational studies in Nutritional Epidemiology (STROBE-Nut) checklist.

| **Section/topic** | **Item number** | **Recommendation** | **Page (line number)** |
| --- | --- | --- | --- |
| Title and abstract | 1 | Indicate the study’s design with a commonly used term in the title or the abstract | 1 (2) |
|  |  | Provide in the abstract an informative and balanced summary of what was done and what was found | 2-3 (10-38) |
| Introduction |  |  |  |
| Background/rationale | 2 | Explain the scientific background and rationale for the investigation being reported | 4-5 (39-77) |
| Objectives | 3 | State specific objectives, including any prespecified hypotheses | 5 (74-77) |
| Methods |  |  |  |
| Study design | 4 | Present key elements of study design early in the manuscript | 5-6 (82-96) |
| Setting | 5 | Describe the setting, locations, and relevant dates, including periods of recruitment, exposure, follow-up, and data collection | 5-6 (82-96) |
| Participants | 6 | Cohort study - give the eligibility criteria, and the sources and methods of selection of participants; describe methods of follow-up | 5-6 (82-96); Additional file 1: Fig S1 |
|  |  | Cohort study - for matched studies, give matching criteria and number of exposed and unexposed Case-control study - for matched studies, give matching criteria and the number of controls per case | N/A |
| Variables | 7 | Clearly define all outcomes, exposures, predictors, potential confounders, and effect modifiers; give diagnostic criteria, if applicable | 6-7 (97-118); 7-9 (129-173); Additional file 1: Supplementary methods |
| Data sources/measurement | 8* | For each variable of interest, give sources of data and details of methods of assessment (measurement); describe comparability of assessment methods if there is more than one group | 6-7 (97-118); 7-9 (129-173); Additional file 1: Supplementary methods |
| Bias | 9 | Describe any efforts to address potential sources of bias | 7-8 (129-142) |
| Study size | 10 | Explain how the study size was arrived at | 5-6 (91-96); 10 (186-191); Additional file 1: Fig S1 |
| Quantitative variables | 11 | Explain how quantitative variables were handled in the analyses; if applicable, describe which groupings were chosen and why | 8 (151-155) |
| Statistical methods | 12 | Describe all statistical methods, including those used to control for confounding | 7-9 (119-184) |
|  |  | Describe any methods used to examine subgroups and interactions | 8 (150-155) |
|  |  | Explain how missing data were addressed | 9 (180-184) |
|  |  | Cohort study - if applicable, explain how loss to follow-up was addressed | N/A |
|  |  | Describe any sensitivity analyses | 9 (174-184); Additional file 1: Supplementary methods |
| Results |  |  |  |
| Participants | 13* | Report numbers of individuals at each stage of study - e.g., numbers potentially eligible, examined for eligibility, confirmed eligible, included in the study, completing follow-up, and analyzed | 10 (186-191); Additional file 1: Fig S1 |
|  |  | Give reasons for nonparticipation at each stage Consider use of a flow diagram | 10 (186-191); Additional file 1: Fig S1 |
| Descriptive data | 14* | Give characteristics of study participants (e.g., demographic, clinical, social) and information on exposures and potential confounders | 10 (192-209); Table 1 |
|  |  | Indicate number of participants with missing data for each variable of interest Cohort study - summarize follow-up time (e.g., average and total amount) | 10 (186-191; 194-195); Additional file 1; Fig S1 |
| Outcome data | 15* | Cohort study - report numbers of outcome events or summary measures over time | 10 (194-195); Fig 1; Table 2 |
| Main results | 16 | Give unadjusted estimates and, if applicable, confounder-adjusted estimates and their precision (e.g., 95% confidence interval); make clear which confounders were adjusted for and why they were included Report category boundaries when continuous variables were categorized | 12 (212-216); Fig 1; Additional file 1: Supplementary methods |
|  |  | If relevant, consider translating estimates of relative risk into absolute risk for a meaningful time period | 12 (215-216); Table 2 |
| Other analyses | 17 | Report other analyses done - e.g., analyses of subgroups and interactions, and sensitivity analyses | 15-19 (260-305); Tables 3, 4, S9, and Fig S6 |
| Discussion |  |  |  |
| Key results | 18 | Summarize key results with reference to study objectives | 19 (307-314) |
| Limitations | 19 | Discuss limitations of the study, taking into account sources of potential bias or imprecision; discuss both direction and magnitude of any potential bias | 22-23 (385-418) |
| Interpretation | 20 | Give a cautious overall interpretation of results considering objectives, limitations, multiplicity of analyses, results from similar studies, and other relevant evidence | 20-22 (333-384) |
| Generalizability | 21 | Discuss the generalizability (external validity) of the study results | 23 (416-418) |
| Other information |  |  |  |
| Funding | 22 | Give the source of funding and the role of the funders for the present study and, if applicable, for the original study on which the present article is based | 25 (437-439) |

### Table S2: Diet group categorisation and definitions.

| **Diet group** | **Definition** |
| --- | --- |
| Regular meat-eater | Total meat intake ≥ 5 servings/week |
| Occasional meat-eater | Total meat intake < 5 servings/week & ≥ 1 serving/month |
| Pescatarian | Total meat intake < 1 serving/month & total fish intake ≥ 1 serving/month |
| Vegetarian | Total meat and fish intakes < 1 serving/month, intake of dairy products or eggs ≥ 1 serving/month |
| Vegan | Total meat, total fish, dairy products, and eggs intake < 1 serving/month |

Diet groups were defined as in Webster et al. (2022) (8).

### Table S3: Summary of mediation analyses using the inverse odds ratio weighting method in the UK Biobank.

| **Step 1**  Determine if mediators differ between diet groups | Apply multiple linear regression models adjusted for confounders for each potential mediator (IV = diet group; DV = potential mediator) |
| --- | --- |
| **Step 2**  Create inverse odds ratio weights | Apply a logistic regression model adjusted for confounders for each mediator (IV = mediator; DV = diet group (binary))  For each mediator, create inverse odds ratio weights for each participant by taking the inverse of the predicted odds ratio for the binary diet groups. Regular meat-eaters (reference group) were assigned a weight of 1, and vegetarians were assigned the inverse odds ratio weights |
| **Step 3**  Calculate total effects | The total effect of the exposure (diet group, binary) on the outcome (hip fracture), conditioning on potential confounders, was estimated using adjusted Cox regression, with age as the underlying time-variable. This is the main Cox model in Fig 1 |
| **Step 4**  Calculate direct effects | The direct effect (i.e. the effect of the exposure on the outcome through pathways besides the mediator of interest) was estimated using adjusted Cox regression as in Step 3, but with the inverse odds ratio weights calculated in Step 2 applied |
| **Step 5**  Calculate indirect effects | The indirect effect (i.e. the effect of the exposure on the outcome through the mediator of interest only) was calculated as the HR or 95% CI for the total effect divided by the HR or 95% CI for the direct effect |
| **Step 6**  Calculate % mediation | % mediation (i.e. the proportion of an association explained by the mediator of interest) was calculated as: [[ln(HR total) – ln(HR direct)] divided by ln(HR total)], multiplied by 100 |
| **Step 7**  Estimate confidence intervals | HRs for total, direct, and indirect effects, as well as % mediation estimates, were bootstrapped to estimate confidence intervals. 300 replications were applied |

HR: hazard ratio. CI: 95% confidence intervals. IV: independent variable. DV: dependent variable.

### Table S4: Diet group classifications at recruitment and at the latest point of available follow-up in UK Biobank participants.

|  |  | | **Diet group at recruitment** | | | | |
| --- | --- | --- | --- | --- | --- | --- | --- |
|  | **Total** | **Regular meat-eater** | | **Occasional meat-eater** | **Pescatarian** | **Vegetarian** | **Vegan** |
| n participants (%) | 57,730 | 35801 (62.0) | | 19009 (32.9) | 1621 (2.8) | 1223 (2.1) | 76 (0.1) |
| **Diet group at latest point of available follow-up** |  |  | |  |  |  |  |
| Regular meat-eater (%) | 34,284 (59.4) | 27,277 (76.2) | | 6,966 (36.6) | 32 (2.0) | 9 (0.7) | 0 (0.0) |
| Occasional meat-eater (%) | 19,713 (34.1) | 8,043 (22.5) | | 11,487 (60.4) | 157 (9.7) | 25 (2.0) | 1 (1.3) |
| Pescatarian (%) | 1,896 (3.3) | 171 (0.5) | | 336 (1.8) | 1,306 (80.6) | 81 (6.6) | 2 (2.6) |
| Vegetarian (%) | 1,673 (2.9) | 292 (0.8) | | 199 (1.0) | 105 (6.5) | 1,058 (86.5) | 19 (25.0) |
| Vegan (%) | 164 (0.3) | 18 (0.1) | | 21 (0.1) | 21 (1.3) | 50 (4.1) | 54 (71.1) |

### Table S5: Dietary characteristics of UK Biobank participants by diet group at recruitment.

| **Dietary food or nutrient intake** | **Total** | **Regular meat-eater** | **Occasional meat-eater** | **Pescatarian** | **Vegetarian** |
| --- | --- | --- | --- | --- | --- |
| **From FFQ, n participants** | 403,968 | 252,126 | 134,940 | 9,435 | 7,467 |
| Poultry (servings/day) | 1.9 (1.2) | 2.5 (1.1) | 1.2 (0.8) | 0.0 (0.1) | 0.0 (0.1) |
| Beef (servings/day) | 0.9 (0.8) | 1.1 (0.9) | 0.6 (0.4) | 0.0 (0.1) | 0.0 (0.0) |
| Lamb (servings/day) | 0.6 (0.5) | 0.7 (0.6) | 0.5 (0.3) | 0.0 (0.0) | 0.0 (0.0) |
| Pork (servings/day) | 0.6 (0.6) | 0.7 (0.6) | 0.5 (0.3) | 0.0 (0.0) | 0.0 (0.0) |
| Processed meat (servings/day) | 1.5 (1.4) | 2.0 (1.4) | 0.7 (0.5) | 0.0 (0.1) | 0.0 (0.1) |
| Oily fish (servings/day) | 1.1 (1.0) | 1.1 (1.0) | 1.2 (1.1) | 1.7 (1.4) | 0.0 (0.1) |
| Non-oily fish (servings/day) | 1.2 (0.9) | 1.2 (0.9) | 1.1 (0.9) | 1.5 (1.2) | 0.0 (0.1) |
| Tea (servings/day) | 3.4 (2.8) | 3.4 (2.8) | 3.3 (2.7) | 3.3 (2.8) | 3.1 (2.9) |
| Coffee (servings/day) | 2.0 (2.0) | 2.1 (2.1) | 1.9 (2.0) | 1.8 (1.9) | 1.7 (2.0) |
| Fruit (servings/day) | 2.7 (1.9) | 2.5 (1.9) | 2.8 (2.0) | 3.3 (2.1) | 3.3 (2.5) |
| Vegetables (servings/day) | 2.5 (1.6) | 2.4 (1.6) | 2.5 (1.7) | 3.1 (2.2) | 3.2 (2.2) |
| **From 24h recall, n participants** | 181,142 | 110,875 | 60,910 | 5,224 | 4,133 |
| Wholegrains (g/day) | 97.0 (90.9) | 92.2 (88.8) | 102.4 (92.4) | 120.1 (98.7) | 117.0 (100.7) |
| Nuts and legumes (servings/day) | 0.4 (0.6) | 0.4 (0.6) | 0.4 (0.6) | 0.6 (0.7) | 0.7 (0.8) |
| SSB's (ml/day) | 4.1 (20.4) | 1.5 (12.0) | 3.6 (17.8) | 29.8 (50.0) | 47.0 (60.6) |
| Fruit juice (ml/day) | 162.8 (277.2) | 178.9 (290.9) | 137.4 (251.2) | 130.9 (251.3) | 145.9 (264.1) |
| Meat substitutes (g/day) | 106.7 (145.4) | 105.6 (145.0) | 106.9 (144.3) | 118.4 (152.8) | 120.9 (160.7) |
| Milk (ml/day) | 154.9 (141.4) | 161.7 (142.7) | 148.3 (138.9) | 122.5 (135.8) | 108.9 (130.2) |
| Cream (g/day) | 1.2 (4.6) | 1.2 (4.7) | 1.2 (4.5) | 1.0 (4.3) | 0.8 (3.7) |
| Dairy desserts (g/day) | 23.7 (46.2) | 24.9 (47.4) | 22.6 (44.9) | 17.6 (38.2) | 18.8 (39.8) |
| Cheese (g/day) | 17.4 (21.0) | 16.6 (20.7) | 17.5 (20.8) | 24.6 (23.6) | 26.8 (25.7) |
| Yoghurt (g/day) | 43.0 (59.1) | 40.9 (58.3) | 46.2 (60.2) | 48.4 (61.6) | 44.7 (60.6) |
| Eggs (g/day) | 21.4 (40.3) | 22.0 (40.9) | 20.3 (39.3) | 23.4 (41.4) | 19.2 (38.2) |
| Protein (g/day) | 81.2 (25.1) | 84.8 (25.5) | 76.9 (23.3) | 68.8 (21.2) | 63.1 (21.2) |
| Protein (g/kg BW/d) | 1.1 (0.4) | 1.1 (0.4) | 1.1 (0.4) | 1.0 (0.3) | 0.9 (0.3) |
| Protein |  |  |  |  |  |
| < 0.75 g/kg BW/d | 29,571 (16.3) | 16,377 (14.8) | 10,768 (17.7) | 1,113 (21.3) | 1,313 (31.8) |
| ≥ 0.75 g/kg BW/d | 151,571 (83.7) | 94,498 (85.2) | 50,142 (82.3) | 4,111 (78.7) | 2,820 (68.2) |
| Protein |  |  |  |  |  |
| < 1.2 g/kg BW/d | 123,877 (68.4) | 73,566 (66.4) | 42,778 (70.2) | 4,052 (77.6) | 3,481 (84.2) |
| ≥ 1.2 g/kg BW/d | 57,265 (31.6) | 37,309 (33.6) | 18,132 (29.8) | 1,172 (22.4) | 652 (15.8) |
| Animal protein (g/day) | 52.8 (21.7) | 56.8 (21.6) | 49.2 (19.7) | 33.6 (16.5) | 23.1 (13.4) |
| Vegetable protein (g/day) | 28.4 (10.6) | 28.0 (10.0) | 27.7 (10.3) | 35.2 (13.5) | 40.0 (16.1) |
| Total carotenoids (µg/day) | 2983 (2823) | 2916 (2748) | 3022 (2874) | 3486 (3244) | 3596 (3279) |
| alpha carotene (µg/day) | 518.7 (645.2) | 517.6 (643.2) | 516.6 (645.9) | 537.4 (664.2) | 555.2 (661.6) |
| beta carotene (µg/day) | 2637 (2444) | 2575 (2375) | 2672 (2492) | 3097 (2822) | 3192 (2865) |
| beta cryptoxanthin (µg/day) | 174.6 (380.6) | 163.7 (359.1) | 183.2 (398.0) | 240.5 (480.1) | 253.3 (497.0) |
| Calcium (mg/day) | 987.8 (348.7) | 990.9 (349.9) | 970.2 (340.3) | 1054 (365.8) | 1081 (387.5) |
| Carbohydrates (g/day) | 255.5 (81.0) | 258.7 (82.4) | 248.1 (77.8) | 259.9 (76.5) | 272.6 (86.3) |
| Starch (g/day) | 129.7 (49.9) | 132.4 (50.4) | 123.8 (47.9) | 132.5 (50.0) | 142.6 (56.3) |
| Fibre (g/day) | 17.9 (6.8) | 17.6 (6.6) | 18.0 (6.8) | 21.2 (7.4) | 22.6 (8.7) |
| Sugar (g/day) | 61.2 (36.8) | 63.6 (38.3) | 57.5 (34.1) | 55.1 (31.4) | 58.0 (34.1) |
| Energy (kcal/day) | 8695 (2537) | 8924 (2593) | 8318 (2384) | 8408 (2370) | 8485 (2674) |
| Fat (g/day) | 73.6 (29.2) | 75.8 (29.9) | 69.9 (27.6) | 72.1 (28.5) | 73.4 (31.1) |
| Animal fat (g/day) | 41.3 (20.2) | 43.5 (20.6) | 38.6 (18.8) | 33.4 (18.4) | 30.6 (19.4) |
| MUFA (g/day) | 26.7 (11.3) | 27.6 (11.5) | 25.2 (10.6) | 25.8 (11.0) | 26.3 (12.1) |
| PUFA (g/day) | 874.2 (393.4) | 859.3 (373.6) | 894.4 (424.3) | 1012.4 (470.7) | 804.1 (257.3) |
| SFA (g/day) | 27.2 (12.3) | 28.1 (12.5) | 25.9 (11.6) | 25.4 (11.7) | 25.8 (12.5) |
| Omega-3 fatty acids (mg/day) | 2000 (1014) | 2018 (1002) | 1959 (1029) | 2237 (1143.5) | 1823 (838.5) |
| Omega-6 fatty acids (g/day) | 11.0 (5.2) | 11.2 (5.2) | 10.5 (5.1) | 12.1 (5.7) | 13.1 (6.5) |
| Trans fats (g/day) | 1.2 (0.7) | 1.2 (0.7) | 1.1 (0.6) | 1.1 (0.7) | 1.2 (0.7) |
| Iron (mg/day) | 12.4 (3.9) | 12.6 (3.9) | 12.1 (3.8) | 12.9 (4.1) | 13.3 (4.7) |
| Haem iron (mg/day) | 0.6 (0.5) | 0.7 (0.5) | 0.5 (0.5) | 0.2 (0.2) | 0.0 (0.1) |
| Iodine (µg/day) | 211.3 (102.9) | 215.2 (103.9) | 207.9 (101.7) | 208.2 (107.4) | 161.0 (68.7) |
| Magnesium (mg/day) | 334.7 (97.4) | 335.5 (96.6) | 328.8 (95.9) | 361.7 (103.8) | 366.3 (119.7) |
| Manganese (mg/day) | 4.2 (1.5) | 4.2 (1.5) | 4.3 (1.5) | 5.1 (1.7) | 5.4 (2.0) |
| Niacin (mg/day) | 38.3 (12.1) | 40.0 (12.2) | 36.3 (11.3) | 32.3 (10.0) | 29.3 (9.7) |
| Phosphorus (mg/day) | 1433 (401.8) | 1461 (405.3) | 1389 (389.4) | 1403 (395.7) | 1376 (427.5) |
| Potassium (mg/day) | 3682 (1094) | 3721 (1095) | 3609 (1079) | 3709 (1084) | 3663 (1214) |
| Retinol (µg/day) | 474.3 (925.2) | 498.1 (979.5) | 451.2 (887.0) | 345.8 (251.3) | 338.9 (202.7) |
| Riboflavin (mg/day) | 1.9 (0.6) | 1.9 (0.6) | 1.9 (0.6) | 1.9 (0.7) | 2.0 (0.9) |
| Selenium (µg/day) | 52.5 (24.6) | 54.0 (24.6) | 51.2 (24.5) | 52.2 (26.1) | 34.8 (14.5) |
| Sodium (mg/day) | 1971 (792.5) | 2038 (819.6) | 1851 (730.9) | 1950 (733.4) | 1962 (785.7) |
| Thiamine (mg/day) | 1.9 (0.7) | 1.9 (0.7) | 1.8 (0.7) | 2.1 (1.1) | 2.3 (1.2) |
| Nitrogen (g/day) | 12.7 (3.9) | 13.2 (4.0) | 12.0 (3.6) | 11.2 (3.5) | 10.4 (3.5) |
| Folate (µg/day) | 313.9 (109.9) | 314.4 (108.9) | 307.6 (108.0) | 341.3 (118.9) | 358.1 (134.5) |
| Vitamin B12 (µg/day) | 6.2 (3.4) | 6.4 (3.4) | 6.1 (3.3) | 5.6 (3.1) | 3.7 (2.0) |
| Vitamin B6 (mg/day) | 2.1 (0.7) | 2.1 (0.7) | 2.0 (0.7) | 1.9 (0.6) | 1.8 (0.7) |
| Vitamin C (mg/day) | 128.4 (78.9) | 126.0 (77.7) | 130.3 (79.6) | 144.0 (82.1) | 147.4 (90.4) |
| Vitamin D (µg/day) | 3.6 (2.9) | 3.7 (2.8) | 3.6 (3.0) | 3.8 (3.4) | 2.1 (1.6) |
| Zinc (mg/day) | 9.8 (3.4) | 10.1 (3.5) | 9.3 (3.2) | 8.6 (2.8) | 9.0 (3.3) |

Participants with missing dietary data were excluded. FFQ: food frequency questionnaire. SSB: sugar-sweetened beverages; BW: body weight; MUFA: mono-unsaturated fatty acids; PUFA: polyunsaturated fatty acids; SFA: saturated fatty acids.

### Table S6: Characteristics of UK Biobank participants by diet group at recruitment, stratified by sex.

| **Characteristics, mean (SD), or n (%)** | **Male** | | | | **Female** | | | |
| --- | --- | --- | --- | --- | --- | --- | --- | --- |
|  | **Regular meat-eater** | **Occasional meat-eater** | **Pescatarian** | **Vegetarian** | **Regular meat-eater** | **Occasional meat-eater** | **Pescatarian** | **Vegetarian** |
| Participants (%) | 139,354 (69.8) | 54,842 (27.5) | 2811 (1.4) | 2,681 (1.3) | 119,411 (55.7) | 83,112 (38.8) | 6746 (3.1) | 4957 (2.3) |
| Cases (%) | 883 (0.6) | 381 (0.7) | 19 (0.7) | 24 (0.9) | 1162 (1.0) | 929 (1.1) | 59 (0.9) | 46 (0.9) |
| **Socio-demographics** |  |  |  |  |  |  |  |  |
| Age, years (SD) | 56.4 (8.2) | 57.6 (8.0) | 54.3 (8.0) | 53.1 (8.0) | 55.7 (8.1) | 56.5 (7.9) | 53.7 (8.0) | 52.8 (7.9) |
| Sex (%) |  |  |  |  |  |  |  |  |
| Male | 123,616 (88.7) | 48,870 (89.1) | 2524 (89.8) | 2454 (91.5) | 105,309 (88.2) | 73,622 (88.6) | 6057 (89.8) | 4512 (91.0) |
| Female | 9998 (7.2) | 3595 (6.6) | 172 (6.1) | 137 (5.1) | 9132 (7.6) | 6021 (7.2) | 403 (6.0) | 251 (5.1) |
| Region (%) | 5740 (4.1) | 2377 (4.3) | 115 (4.1) | 90 (3.4) | 4970 (4.2) | 3469 (4.2) | 286 (4.2) | 194 (3.9) |
| England |  |  |  |  |  |  |  |  |
| Scotland | 133,264 (95.6) | 51,817 (94.5) | 2620 (93.2) | 2176 (81.2) | 113,948 (95.4) | 78,963 (95.0) | 6357 (94.2) | 4106 (82.8) |
| Wales | 2002 (1.4) | 603 (1.1) | 43 (1.5) | 11 (0.4) | 2107 (1.8) | 1221 (1.5) | 95 (1.4) | 31 (0.6) |
| Ethnicity (%) | 2362 (1.7) | 1722 (3.1) | 100 (3.6) | 457 (17.0) | 1608 (1.3) | 1531 (1.8) | 185 (2.7) | 727 (14.7) |
| White | 677 (0.5) | 224 (0.4) | 21 (0.7) | 13 (0.5) | 768 (0.6) | 590 (0.7) | 63 (0.9) | 46 (0.9) |
| Black | 1049 (0.8) | 476 (0.9) | 27 (1.0) | 24 (0.9) | 980 (0.8) | 807 (1.0) | 46 (0.7) | 47 (0.9) |
| Asian | 46,611 (46.3) | 20,310 (51.4) | 1663 (72.6) | 1469 (68.8) | 35918 (42.5) | 29236 (48.9) | 3611 (66.9) | 2456 (63.4) |
| Mixed | -1.4 (3.1) | -1.4 (3.1) | -0.8 (3.2) | -0.6 (3.2) | -1.5 (3.0) | -1.3 (3.0) | -1.1 (3.0) | -0.8 (3.0) |
| Other | 22,319 (16.0) | 10,124 (18.5) | 629 (22.4) | 551 (20.6) | 19,087 (16.0) | 19,806 (23.8) | 1658 (24.6) | 1071 (21.6) |
| Degree-level education (%) |  |  |  |  |  |  |  |  |
| Townsend deprivation index (SD) | 3253 (4502) | 3102 (4170) | 3201 (4007) | 2940 (3731) | 2671 (3274) | 2741 (3325) | 2970 (3372) | 2872 (3668) |
| Live alone (%) |  |  |  |  |  |  |  |  |
| **Lifestyle** | 17,835 (12.8) | 5848 (10.7) | 233 (8.3) | 240 (9.0) | 10,481 (8.8) | 7340 (8.8) | 443 (6.6) | 277 (5.6) |
| Physical activity, METs.mins/week (SD) | 53,722 (38.6) | 21,134 (38.5) | 1031 (36.7) | 897 (33.5) | 37,028 (31.0) | 26256 (31.6) | 2406 (35.7) | 1389 (28.0) |
| Smoking status (%) | 67,797 (48.7) | 27,860 (50.8) | 1547 (55.0) | 1544 (57.6) | 71,902 (60.2) | 49,516 (59.6) | 3897 (57.8) | 3291 (66.4) |
| Current | 1.7 (1.8) | 1.4 (1.5) | 1.3 (1.4) | 1.0 (1.5) | 0.9 (1.1) | 0.8 (1.0) | 0.8 (1.0) | 0.6 (1.0) |
| Former | 60,761 (43.6) | 25,513 (46.5) | 1387 (49.3) | 1235 (46.1) | 63,627 (53.3) | 47,091 (56.7) | 3985 (59.1) | 2843 (57.4) |
| Never |  |  |  |  |  |  |  |  |
| Alcohol consumption (drinks/day) | 28.1 (4.3) | 27.3 (4.0) | 25.8 (3.6) | 25.8 (3.9) | 27.5 (5.2) | 26.4 (4.8) | 25.0 (4.5) | 25.5 (4.9) |
| Nutritional supplementation (%) | 295 (0.2) | 115 (0.2) | 14 (0.5) | 18 (0.7) | 660 (0.6) | 731 (0.9) | 135 (2.0) | 102 (2.1) |
| **Anthropometrics** | 32,023 (23.0) | 15,805 (28.8) | 1273 (45.3) | 1207 (45.0) | 42,783 (35.8) | 36,806 (44.3) | 3765 (55.8) | 2568 (51.8) |
| BMI, kg/m2 (SD) | 107,036 (76.8) | 38,922 (71.0) | 1524 (54.2) | 1456 (54.3) | 75,968 (63.6) | 45,575 (54.8) | 2846 (42.2) | 2287 (46.1) |
| Height, m (SD) | 175.8 (6.8) | 175.6 (6.8) | 176.4 (6.8) | 175.6 (7.2) | 162.6 (6.2) | 162.7 (6.3) | 163.7 (6.3) | 162.5 (6.8) |
| **Comorbidities** |  |  |  |  |  |  |  |  |
| Prevalence of diabetes (%) | 16,660 (12.0) | 5996 (10.9) | 158 (5.6) | 253 (9.4) | 8502 (7.1) | 4863 (5.9) | 237 (3.5) | 301 (6.1) |
| Prevalence of cancer (%) | 10,971 (7.9) | 4555 (8.3) | 187 (6.7) | 119 (4.4) | 14,817 (12.4) | 10,666 (12.8) | 814 (12.1) | 512 (10.3) |
| Prevalence of CVD (%) | 19,693 (14.1) | 8129 (14.8) | 244 (8.7) | 232 (8.7) | 10,436 (8.7) | 6724 (8.1) | 365 (5.4) | 272 (5.5) |
| Prevalence of other fracture (%) | 13,577 (9.7) | 4733 (8.6) | 314 (11.2) | 256 (9.5) | 12,223 (10.2) | 8827 (10.6) | 712 (10.6) | 554 (11.2) |
| **Female-specific covariates** | N/A | N/A | N/A | N/A |  |  |  |  |
| Menopausal status (%) | N/A | N/A | N/A | N/A |  |  |  |  |
| Premenopausal | N/A | N/A | N/A | N/A | 36,214 (30.3) | 21,389 (25.7) | 2516 (37.3) | 2043 (41.2) |
| Postmenopausal | N/A | N/A | N/A | N/A | 83,197 (69.7) | 61,723 (74.3) | 4230 (62.7) | 2914 (58.8) |
| HRT use (%) | N/A | N/A | N/A | N/A |  |  |  |  |
| Current | N/A | N/A | N/A | N/A | 7385 (6.2) | 5111 (6.1) | 394 (5.8) | 212 (4.3) |
| Former | N/A | N/A | N/A | N/A | 33,525 (28.1) | 24,129 (29.0) | 1331 (19.7) | 773 (15.6) |
| Never | N/A | N/A | N/A | N/A | 78,501 (65.7) | 53,872 (64.8) | 5021 (74.4) | 3972 (80.1) |
| ≥ 1 children (%) | N/A | N/A | N/A | N/A | 99,652 (83.5) | 65,071 (78.3) | 4673 (69.3) | 3431 (69.2) |

### Table S7: Characteristics of UK Biobank participants at recruitment that were included or excluded from analyses.

| **Characteristics, mean (SD) or n (%)** | **489,703 potentially eligible participants** | | | | **75,789 participants excluded due to missing covariate data** | | | |
| --- | --- | --- | --- | --- | --- | --- | --- | --- |
|  | **Regular meat-eater** | **Occasional meat-eater** | **Pescatarian** | **Vegetarian** | **Regular meat-eater** | **Occasional meat-eater** | **Pescatarian** | **Vegetarian** |
| Participants (%) | 304,576 (62.2) | 164,591 (33.6) | 11,104 (2.3) | 9,432 (1.9) | 45,811 (60.4) | 26,637 (35.1) | 1,547 (2.0) | 1,794 (2.4) |
| Cases (%) | 2588 (0.8) | 1644 (1.0) | 101 (0.9) | 85 (0.9) | 543 (1.2) | 334 (1.3) | 23 (1.5) | 15 (0.8) |
| **Socio-demographics** |  |  |  |  |  |  |  |  |
| Age, years (SD) | 56.3 (8.1) | 57.2 (7.9) | 54.1 (8.0) | 53.3 (8.0) | 57.7 (7.9) | 58.5 (7.6) | 55.6 (7.9) | 55.0 (8.1) |
| Sex (%) |  |  |  |  |  |  |  |  |
| Male | 155,610 (51.1) | 61,532 (37.4) | 3086 (27.8) | 3193 (33.9) | 16,256 (35.5) | 6690 (25.1) | 275 (17.8) | 512 (28.5) |
| Female | 148,966 (48.9) | 103,059 (62.6) | 8018 (72.2) | 6239 (66.1) | 29,555 (64.5) | 19,947 (74.9) | 1272 (82.2) | 1282 (71.5) |
| Region (%) |  |  |  |  |  |  |  |  |
| England | 269,451 (88.5) | 146,172 (88.8) | 9978 (89.9) | 8631 (91.5) | 40,526 (88.5) | 23,680 (88.9) | 1397 (90.3) | 1665 (92.8) |
| Scotland | 22,586 (7.4) | 11,401 (6.9) | 657 (5.9) | 464 (4.9) | 3456 (7.5) | 1785 (6.7) | 82 (5.3) | 76 (4.2) |
| Wales | 12,539 (4.1) | 7,018 (4.3) | 469 (4.2) | 337 (3.6) | 1829 (4.0) | 1172 (4.4) | 68 (4.4) | 53 (3.0) |
| Ethnicity (%) |  |  |  |  |  |  |  |  |
| White | 289,386 (95.3) | 155,336 (94.7) | 10,344 (93.7) | 7147 (80.7) | 42,174 (94.2) | 24,556 (94.0) | 1367 (92.2) | 865 (71.1) |
| Black | 5028 (1.7) | 2302 (1.4) | 168 (1.5) | 46 (0.5) | 919 (2.1) | 478 (1.8) | 30 (2.0) | 4 (0.3) |
| Asian | 4877 (1.6) | 3916 (2.4) | 342 (3.1) | 1508 (17.0) | 907 (2.0) | 663 (2.5) | 57 (3.8) | 324 (26.6) |
| Mixed | 1729 (0.6) | 964 (0.6) | 96 (0.9) | 67 (0.8) | 284 (0.6) | 150 (0.6) | 12 (0.8) | 8 (0.7) |
| Other | 2538 (0.8) | 1550 (0.9) | 89 (0.8) | 87 (1.0) | 509 (1.1) | 267 (1.0) | 16 (1.1) | 16 (1.3) |
| Degree-level education (%) | 92,012 (43.1) | 55,807 (48.3) | 5938 (67.1) | 4401 (63.9) | 9483 (33.7) | 6261 (38.4) | 664 (57.4) | 476 (54.2) |
| Townsend deprivation index (SD) | -1.4 (3.1) | -1.3 (3.1) | -1.0 (3.1) | -0.6 (3.2) | -1.0 (3.2) | -1.0 (3.2) | -0.8 (3.2) | 0.3 (3.5) |
| Live alone (%) | 49,083 (16.2) | 35,966 (22.0) | 2686 (24.4) | 1904 (21.6) | 7677 (17.5) | 6036 (23.7) | 399 (27.6) | 282 (23.7) |
| **Lifestyle** |  |  |  |  |  |  |  |  |
| Physical activity, METs.mins/week (SD) | 2975 (3988) | 2880 (3683) | 3039 (3579) | 2912 (3892) | 2738 (3864) | 2794 (3581) | 3044 (3722) | 3227 (6611) |
| Smoking status (%) |  |  |  |  |  |  |  |  |
| Current | 33,746 (11.1) | 16,026 (9.8) | 800 (7.2) | 611 (6.5) | 5430 (12.1) | 2838 (10.9) | 124 (8.2) | 94 (5.4) |
| Former | 106,287 (35.0) | 56,172 (34.3) | 3963 (35.8) | 2601 (27.7) | 15,537 (34.7) | 8782 (33.8) | 526 (34.8) | 315 (17.9) |
| Never | 163,514 (53.9) | 91,761 (56.0) | 6304 (57.0) | 6181 (65.8) | 23,815 (53.2) | 14,385 (55.3) | 860 (57.0) | 1346 (76.7) |
| Alcohol consumption (drinks/day) | 1.3 (1.5) | 1.0 (1.2) | 0.9 (1.2) | 0.7 (1.2) | 1.0 (1.4) | 0.8 (1.1) | 0.8 (1.1) | 0.4 (0.8) |
| Nutritional supplementation (%) | 147,359 (48.5) | 87,077 (53.0) | 6298 (56.8) | 4789 (50.9) | 22,971 (51.2) | 14,473 (55.2) | 926 (60.4) | 711 (40.4) |
| **Anthropometrics** |  |  |  |  |  |  |  |  |
| BMI, kg/m2 (SD) | 27.9 (4.8) | 26.8 (4.6) | 25.3 (4.3) | 25.8 (4.7) | 28.3 (5.2) | 27.3 (5.0) | 25.7 (4.7) | 26.8 (5.2) |
| Height, m (SD) | 169.2 (9.3) | 167.4 (9.1) | 167.1 (8.7) | 166.5 (9.4) | 166.3 (9.0) | 165.0 (8.5) | 165.0 (8.6) | 163.8 (9.0) |
| **Comorbidities** |  |  |  |  |  |  |  |  |
| Prevalence of diabetes (%) | 30,733 (10.1) | 13,534 (8.2) | 494 (4.4) | 790 (8.4) | 5571 (12.2) | 2675 (10.0) | 99 (6.4) | 236 (13.2) |
| Prevalence of cancer (%) | 31,043 (10.2) | 18,461 (11.2) | 1171 (10.5) | 760 (8.1) | 5255 (11.5) | 3240 (12.2) | 170 (11.0) | 129 (7.2) |
| Prevalence of CVD (%) | 36,856 (12.1) | 18,461 (11.2) | 735 (6.6) | 716 (7.6) | 6727 (14.7) | 3608 (13.5) | 126 (8.1) | 212 (11.8) |
| Prevalence of other fracture (%) | 30,891 (10.1) | 16,455 (10.0) | 1206 (10.9) | 946 (10.0) | 5091 (11.1) | 2895 (10.9) | 180 (11.6) | 136 (7.6) |
| **Female-specific covariates** |  |  |  |  |  |  |  |  |
| Menopausal status (%) |  |  |  |  |  |  |  |  |
| Premenopausal | 41,426 (28.0) | 24,390 (23.8) | 2841 (35.7) | 2323 (38.4) | 5212 (18.3) | 3001 (15.5) | 325 (26.9) | 280 (25.5) |
| Postmenopausal | 106,539 (72.0) | 78,085 (76.2) | 5115 (64.3) | 3733 (61.6) | 23,342 (81.7) | 16,362 (84.5) | 885 (73.1) | 819 (74.5) |
| HRT use (%) |  |  |  |  |  |  |  |  |
| Current | 9117 (6.5) | 6169 (6.4) | 477 (6.2) | 266 (4.4) | 1732 (8.2) | 1058 (7.6) | 83 (8.7) | 54 (5.2) |
| Former | 39,940 (28.4) | 28,412 (29.3) | 1532 (19.9) | 906 (15.1) | 6415 (30.5) | 4283 (30.7) | 201 (21.0) | 133 (12.7) |
| Never | 91,388 (65.1) | 62,498 (64.4) | 5695 (73.9) | 4830 (80.5) | 12,887 (61.3) | 8626 (61.8) | 674 (70.4) | 858 (82.1) |
| ≥ 1 children (%) | 124,995 (84.0) | 81,340 (79.0) | 5566 (69.5) | 4141 (66.5) | 25,343 (86.1) | 16,269 (82.0) | 893 (70.6) | 710 (55.9) |

SD: standard deviation; METs: Metabolic equivalents; BMI: body mass index; CVD: cardiovascular disease; HRT: hormone replacement therapy.

### Table S8: Adjusted and relative means (95% confidence intervals) of potential mediators at recruitment across diet groups in the UK Biobank.

| **Diet group** |  | **Cases / participants** | **Adjusted mean (95% CI)** | **Relative mean (95% CI)** |
| --- | --- | --- | --- | --- |
| **BMI** |  | **3503/413,914** |  |  |
| Regular meat-eater |  | 2045/256,720 | 27.74 (27.72, 27.75) | 1.00 |
| Occasional meat-eater |  | 1310/136,644 | 26.81 (26.78, 26.83) | 0.97 (0.97, 0.97) |
| Pescatarian |  | 78/9479 | 25.56 (25.47, 25.65) | 0.92 (0.92, 0.92) |
| Vegetarian |  | 70/7568 | 25.93 (25.83, 26.03) | 0.93 (0.93, 0.94) |
| **Heel BMD t-score** |  | **3016/364,864** |  |  |
| Regular meat-eater |  | 1768/229,735 | -0.21 (-0.21, -0.20) | 1.00 |
| Occasional meat-eater |  | 1122/122,799 | -0.21 (-0.21, -0.20) | 1.00 (1.00, 1.00) |
| Pescatarian |  | 66/8512 | -0.20 (-0.23, -0.18) | 1.05 (0.90, 1.10) |
| Vegetarian |  | 60/6834 | -0.22 (-0.25, -0.20) | 0.95 (0.81, 1.00) |
| **FFM** |  | **3418/408,176** |  |  |
| Regular meat-eater |  | 1990/253,148 | 54.13 (54.11, 54.15) | 1.00 |
| Occasional meat-eater |  | 1288/134,767 | 53.29 (53.26, 53.31) | 0.98 (0.98, 0.98) |
| Pescatarian |  | 74/9368 | 52.41 (52.31, 52.52) | 0.97 (0.97, 0.97) |
| Vegetarian |  | 66/7475 | 52.48 (52.36, 52.60) | 0.97 (0.97, 0.97) |
| **Hand grip strength** |  | **3483/412,770** |  |  |
| Regular meat-eater |  | 20321/256,050 | 33.39 (33.36, 33.42) | 1.00 |
| Occasional meat-eater |  | 1305/136,247 | 33.25 (33.21, 33.29) | 1.00 (1.00, 1.00) |
| Pescatarian |  | 78/9443 | 33.61 (33.46, 33.76) | 1.01 (1.00, 1.01) |
| Vegetarian |  | 69/7547 | 33.23 (33.06, 33.40) | 1.00 (0.99, 1.00) |
| **Vitamin D** |  | **3093/370,769** |  |  |
| Regular meat-eater |  | 1807/230,331 | 44.3 (44.3, 44.3) | 1.00 |
| Occasional meat-eater |  | 1151/122,150 | 44.3 (44.3, 44.7) | 1.00 (1.00, 1.00) |
| Pescatarian |  | 68/8563 | 42.9 (42.5, 43.4) | 0.99 (0.99, 0.99) |
| Vegetarian |  | 67/6632 | 36.6 (36.2, 37.0) | 0.95 (0.95, 0.95) |
| **IGF-1** |  | **3233/385,653** |  |  |
| Regular meat-eater |  | 1880/239,135 | 21.65 (21.63, 21.67) | 1.00 |
| Occasional meat-eater |  | 1214/127,345 | 21.42 (21.39, 21.45) | 0.99 (0.99, 0.99) |
| Pescatarian |  | 70/8861 | 21.37 (21.26, 21.49) | 0.99 (0.98, 0.99) |
| Vegetarian |  | 69/7079 | 20.39 (20.27, 20.52) | 0.94 (0.94, 0.95) |

Adjusted means were calculated from multiple linear regression models, all of which were adjusted for (all at recruitment): age, region, sex, ethnicity, Townsend Deprivation Index, live alone, smoking status, nutritional supplementation , MET minutes of physical activity per week, alcohol consumption in drinks per day, prevalence of diabetes, cancer, cardiovascular disease, and other non-hip fractures, number of children, menopausal status, and hormone replacement therapy use. Models for heel BMD, hand grip strength, vitamin D and IGF-1 were also adjusted for BMI, whilst the model for FFM was adjusted for height. Relative means were calculated by comparing adjusted means for each diet group with that of regular meat-eaters. BMI: body mass index; FFM: fat-free mass; IGF-1: insulin-like growth factor-1; HR (95% CI): hazard ratio (95% confidence intervals).

### Table S9: Risk of hip fracture by diet group in the UK Biobank with varying restrictions.

| **Diet group** | **Cases/subjects** | **HR (95% CI)** |
| --- | --- | --- |
| **Adjusted model** | 3503/413,914 |  |
| Regular meat-eater (reference) | 2045/258,765 | 1 |
| Occasional meat-eater | 1310/137,954 | 0.99 (0.93, 1.07) |
| Pescatarian | 78/9557 | 1.08 (0.86, 1.35) |
| Vegetarian | 70/7638 | 1.50 (1.18, 1.91) |
| **Adjusted for height and weight instead of BMI** | 3503/413,914 |  |
| Regular meat-eater (reference) | 2045/258,765 | 1 |
| Occasional meat-eater | 1310/137,954 | 0.99 (0.92, 1.06) |
| Pescatarian | 78/9557 | 1.06 (0.85, 1.34) |
| Vegetarian | 70/7638 | 1.49 (1.17, 1.89) |
| **Excluding participants with < 3 years of follow-up** | 3116/410,187 |  |
| Regular meat-eater (reference) | 1803/256,348 | 1 |
| Occasional meat-eater | 1179/136,748 | 1.01 (0.94, 1.09) |
| Pescatarian | 70/9496 | 1.14 (0.90, 1.45) |
| Vegetarian | 64/7595 | 1.64 (1.27, 2.11) |
| **Excluding participants on long-term treatment for illness** | 998/183,711 |  |
| Regular meat-eater (reference) | 558/113,299 | 1 |
| Occasional meat-eater | 377/61262 | 0.98 (0.86, 1.12) |
| Pescatarian | 28/5096 | 1.01 (0.69, 1.48) |
| Vegetarian | 35/4054 | 1.91 (1.35, 2.70) |
| **Vegetarians and vegans separated** | 3503/413,914 |  |
| Regular meat-eater (reference) | 2045/258,765 | 1 |
| Occasional meat-eater | 1310/137,954 | 0.99 (0.93, 1.07) |
| Pescatarian | 78/9557 | 1.08 (0.86, 1.35) |
| Vegetarian | 60/7238 | 1.38 (1.06, 1.79) |
| Vegan | 10/400 | 3.26 (1.75, 6.08) |
| **Accounting for death as a competing risk** | 3503/413,914 |  |
| Regular meat-eater (reference) | 2045/258,765 | 1 |
| Occasional meat-eater | 1310/137,954 | 1.00 (0.93, 1.08) |
| Pescatarian | 78/9557 | 1.06 (0.85, 1.34) |
| Vegetarian | 70/7638 | 1.47 (1.15, 1.88) |

The adjusted model controlled for age, and was adjusted for the following (all at recruitment): region (England, Scotland, Wales), sex (male, female), ethnicity (white, black, Asian, mixed, other), Townsend deprivation index (continuous), live alone (yes, no), smoking (current, former, never), supplementation (yes, no), physical activity (continuous), alcohol consumption (continuous), body mass index (continuous), number of children (0, 1, 2, ≥ 3), menopausal status (premenopausal, postmenopausal), hormone replacement therapy (current, former, never), diabetes (yes, no), cancer (yes, no), cardiovascular disease (yes, no), and other fracture (yes, no). All other models were based on the adjusted model. HR (95% CI): hazard ratio (95% confidence interval). HRT: hormone replacement therapy use at recruitment.

# **Supplementary Methods**

## Diet group classification

At recruitment and in the repeat assessments in 2012-2013, 2014, and in 2019, participants were asked questions on their frequency of intake of oily fish, non-oily fish, processed meat, poultry, beef, lamb/mutton, pork, eggs, and dairy products (22). Questions on meat and fish were asked in the form of “how often do you eat [specific food or beverage?]” or similar. Options were 1 “never”, 2 “less than once a week”, “3 once a week”, 4 “2-4 times a week”, 5 “5-6 times a week”, or 6 “once or more daily”. Responses to these individual questions were converted into weekly-based consumption frequencies as follows: 0, 0.5, 1, 3, 5.5, 7 servings/week. Participants could also answer “do not know” or “prefer not to say” for these questions. Responses to questions on intake of processed meat, poultry, beef, lamb/mutton, and pork were summed to form total meat intake (servings/week); and questions on intake of oily and non-oily fish intake were summed to form total fish intake (servings/week). Intake of eggs and dairy products was assessed by asking participants “Which of the following do you never eat?”, with options of “Eggs or foods containing eggs”, “Dairy products”, “I eat all of the above”, or “Prefer not to answer”.

Participants were then classified as regular meat-eaters (ate meat ≥ 5 times/week), occasional meat-eaters (ate meat < 5 times/week), pescatarians (ate fish but not meat), vegetarians (ate eggs or dairy but not meat or fish), or vegans (did not eat meat, fish, eggs, or dairy) at recruitment and at the latest point of available follow-up in each participant, with vegans combined into the vegetarian group. Diet group classifications at recruitment were used to represent diet group over follow-up. We considered non-responders for each specific question to be non-consumers of that food item, however the final complete-case analysis did not include any non-responders for questions related to meat and fish intake. Participants who answered “do not know” or “prefer not to say” to questions on meat, fish, eggs, or dairy intake were coded as missing, and were excluded from analyses, unless there was sufficient data to classify the participant into a diet group (e.g. if a participant reported consuming ≥ 5 servings/week of a specific type of meat but had missing data for other meat types, that participant could be classified as a regular meat-eater).

## Other dietary measurements

From April 2009 to September 2010, the Oxford WebQ 24h dietary recall was added to assessment centres to provide a more detailed assessment of diet. After that, the WebQ questionnaire was administered online once every 3-4 months from February 2011 until June 2012, resulting in four follow-up instances. In each instance, participants were asked to report the number of portions for each item they consumed over the prior 24 h. These were multiplied by standard portion sizes to calculate daily intake in grams per day for each specific food item at each instance (22). Nutrient intakes were calculated automatically in the WebQ via built-in-algorithms using daily intakes of food items and food composition data from the UK Nutrient Databank – this process is described in detail elsewhere (23). We then summed relevant items (in g/day) together (e.g. for fruit intake, we summed reported intakes of apples and pears, berries, citrus, dried fruit, other fruit, and stewed fruit). In participants who completed at least one 24h recall (n=181,142 after applying the exclusion criteria and excluding participants with missing covariate data), we averaged each participant’s reported intake of each food item, food group, or nutrient intake across all available instances, as suggested in a previous study (23).

## Derivation of potential mediators

**Body mass index (BMI)**

Body weight and standing height were measured at the assessment centre visit at recruitment. BMI was calculated as a participant’s body weight (kg) divided by the square of their height (m).

**Other anthropometric measures**

Bioimpedance was measured at the assessment centre visit at recruitment using the Tanita Bc418ma bioimpedance device, from which body fat percentage, whole-body fat mass, whole-body fat-free mass were estimated.

Hand grip strength (for each hand) was measured using the Jamar Hydraulic hand dynamometer, and the highest score of either hand was used in this study.

Calcaneal bone mineral density (BMD) was measured using a Norland McCue Contact Ultrasound Bone Analyzer, from which a heel BMD t-score was calculated for each participant. In participants with BMD measures for the left and right heels, we used the highest BMD t-score value.

**Biomarkers**

Participants provided blood samples at recruitment, from which serum vitamin D and insulin-like Growth Factor-1 (IGF-1) were measured by Chemiluminescence Immunoassay (CLIA) analysis on a DiaSorin Ltd. LIASON XL. A full description of the biomarker measurements can be found on the UK Biobank website (<https://www.ukbiobank.ac.uk/media/gnkeyh2q/study-rationale.pdf>).

## Derivation of covariates

**Age at recruitment**

Calculated as date of recruitment minus date of birth, truncated to whole year.

**Sex**

Genetic sex as determined from genotyping analysis.

**Region**

At recruitment, participants attended one of 22 assessment centres across the UK. We grouped the centres into three regions as follows: England (Barts, Birmingham, Bristol, Bury, Croydon, Hounslow, Leeds, Liverpool, Manchester, Middlesborough, Newcastle, Nottingham, Oxford, Reading, Sheffield, Stockport, Stoke), Scotland (Edinburgh, Glasgow), and Wales (Cardiff, Swansea, and Wrexham).

**Ethnicity**

At recruitment, participants were asked in the touchscreen questionnaire to select their ethnic group among “White”, “Mixed”, “Asian or Asian British”, “Black or black British”, “Chinese”, “Other ethnic group”, “Do not know”, or “Prefer not to say”. We regrouped participants into the following ethnicity categories: White, Mixed race, Asian, Black, and Other.

**Socio-economic status**

The Townsend deprivation index was used as in index of socio-economic status (as a continuous variable). This variable was previously created in the UK biobank resource based on national census output areas. Each participant was assigned a score corresponding to the output area in which their postcode was located.

**Living alone**

In the touchscreen questionnaire at recruitment, participants were asked “Including yourself, how many people are living together in your household?”. From this, we defined the variable “live alone” (yes, no).

**Physical activity**

Physical activity in total metabolic equivalent task (MET) minutes per week was calculated based on a series of questions that asked about frequency and duration of walking, moderate activity, and vigorous activity.

**Smoking status**

At recruitment, participants were asked in the touchscreen questionnaire “Do you smoke tobacco now?” and “In the past, how often have you smoked tobacco?” to determine their smoking status as current, previous, or never.

**Alcohol consumption**

In the touchscreen questionnaire at recruitment, participants were asked about their weekly and monthly intake of glasses of red wine, glasses of champagne plus white wine, pints of beer plus cider, measures of spirits or liqueurs, glasses of fortified wine, and glasses of other alcohol. Participants could input any number, “do not know”, or “prefer not to say”. We summed participants weekly and monthly alcohol intakes, respectively. Weekly total alcohol intake was converted into daily total alcohol intake (drinks/day). Non-responders for weekly and monthly intake of specific alcohol types were considered non-consumers of that specific alcohol type. For participants who answered “do not know” or “prefer not to say” for any question on weekly alcohol intake, monthly intakes were used, if available. Otherwise, we used responses to a question in the touchscreen questionnaire at recruitment that asked “how often do you drink alcohol?” with options of 1 “daily or almost daily”, 2 “three or four times a week”, 3 “once or twice a week”, 4 “one to three times a month”, 5 “special occasions only”, 6 “never”, or 7 “prefer not to answer”.

**Nutritional supplementation**

Participants were asked in the touchscreen questionnaire at recruitment to select which, if any, of the following supplements they consume regularly: vitamin A, vitamin B, vitamin C, vitamin D, vitamin E, folic acid or folate, multivitamins/minerals, fish oil, glucosamine, calcium, zinc, iron, selenium, none of the above, or prefer not to answer. Participants who reported consuming ≥ 1 type of supplement were classified as supplement users. Non-responders and participants who responded “none of the above” were coded as not taking any supplements.

**Comorbidities**

We identified prevalence of hip fracture (yes, no), osteoporosis (yes, no), other non-hip fractures (yes, no), diabetes (yes, no), cardiovascular disease (yes, no), and cancer (yes, no) at recruitment using self-reported information from questions on health and medical history asked in the touchscreen questionnaire, and through use of hospital records and cancer registries (with the date of diagnosis being before or on the date of recruitment).

**Number of children**

At recruitment in the touchscreen questionnaire, women were asked “how many children have you given birth to?”. We grouped responses as 0, 1, 2, 3, or ≥ 4 children.

**Menopausal status**

At recruitment in the touchscreen questionnaire, women were asked multiple questions relating to menopausal status. Women were defined as premenopausal or postmenopausal at recruitment using the following criteria:

**Premenopausal:** answered “no” to the question that asked about having gone through menopause, or answered “not sure”, and:

- Were < 55 years old, did not report having a bilateral oophorectomy or hysterectomy, and did not report using hormone replacement therapy (HRT), or;
- Were < 55 years old, did not report having a bilateral oophorectomy or hysterectomy, and reported menstruating on the day of recruitment.

**Postmenopausal:** answered “yes” to having gone through menopause, or answered “not sure”, and were ≥ 55 years old or had a bilateral oophorectomy.

**Hormone replacement therapy (HRT)**

At recruitment in the touchscreen questionnaire, women were asked “Have you ever used hormone replacement therapy?” and if yes, “How old were you when you last used hormone replacement therapy?” We categorised HRT use based on these questions as “Current”, “Former”, “Never”.

## Calculating absolute risk differences

To determine if any relative risk differences between diet groups are clinically significant, we calculated the absolute risk difference between each diet group and regular meat-eaters as the difference between the predicted incidence per 1000 people over 10 years per diet group. Predicted incidences were calculated using hazard ratios (HR) and 95% confidence intervals (95% CI’s) expressed as floating absolute risks, which assign a 95% CI to all groups including the reference group, allowing estimation of precision for the predicted incidence in regular meat-eaters without arbitrarily assigning a diet group as the reference group (7, 25, 26). Predicted incidence in regular meat-eaters per 1000 people over 10 years was calculated as (1 – Sr) x 1000, where Sr = (1 – observed incidence in regular meat-eaters)^10^, representing the predicted 10-year non-incidence (survival) rate in regular meat-eaters. Predicted incidence in occasional meat-eaters, pescatarians, and vegetarians over the same timeframe was calculated as (1-SR^HR or 95% CI^) x 1000, where HR or 95% CI represents the adjusted hazard ratio or confidence intervals for hip fracture risk per diet group compared to regular meat-eaters, and Sr^HR or 95% CI^ represents the predicted 10-year non-incidence (survival) rate in each diet group compared to regular meat-eaters, after accounting for potential confounders.

## Mediation analyses

All steps of mediation analyses are summarised in Table S3. Mediation analyses were only conducted if a) an association was observed between a diet group and risk of hip fracture, and b) a significant difference in the mediator of interest was observed between a given diet group and regular meat-eaters.

To determine if body mass index (BMI), heel bone mineral density (BMD), fat-free mass (FFM), hand grip strength, serum vitamin D, and insulin-like growth factor-1 (IGF-1) significantly differed between diet groups at recruitment, we used a multivariable linear regression model for each mediator (independent variable = diet group, dependent variable = mediator of interest). All models were adjusted for (all at recruitment): age, region, sex, ethnicity, Townsend Deprivation Index, live alone, smoking status, any nutritional supplementation, MET minutes of physical activity per week, alcohol consumption in drinks per day, prevalence of diabetes, cancer, cardiovascular disease, and other non-hip fractures, number of children, menopausal status, and hormone replacement therapy use. Models for heel BMD, hand grip strength, serum vitamin D and IGF-1 were also adjusted for BMI, whilst the model for FFM was further adjusted for height.

The inverse odds ratio weighting (IORW) method was used to estimate mediation of any significant diet group – hip fracture associations through each of the aforementioned potential mediators. This method leverages the invariance property of an odds ratio (OR; the OR for the relationship between the exposure and mediator is the same regardless of which variable is defined as dependent or independent) to condense the relationship between the exposure and any number of mediators of interest into a single OR, conditional on covariates, by regressing the exposure on the mediator(s) and covariates (4, 27, 28). The inverse of the covariate-adjusted exposure-mediator OR can then be applied as a weight in main regression analyses of the outcome on the exposure. By using mediators to construct weights, mediators are never entered into Cox regression models with the outcome, meaning that the exposure and mediator(s) remain independent (28). The resulting weighted Cox regression model thereby estimates the natural direct effect (NDE) of the exposure on the outcome when the mediating pathway(s) of interest is/are deactivated. The natural indirect effect (NIE) (the exposure – outcome association only through mediator(s) pathways of interest) can then be calculated as the total effect minus the NDE. The IORW method can be applied to Cox regression, accommodates use of multiple mediators of a categorical, discrete, or continuous nature, and is agnostic of exposure-mediator interactions (28). Key assumptions made include: no unmeasured exposure-mediator, mediator-outcome, or exposure-outcome confounding; and no unmeasured mediator-outcome confounding that could be affected by the exposure (4, 27, 28).

We ran the IORW method separately for each mediator (all continuous variables). In each case, weights for each mediator were estimated from logistic regression models adjusted for relevant confounders (as in multivariable linear regression models from step 1) where the binary diet group (regular meat-eater or vegetarian) was the dependent variable, and the mediator was the independent variable. For each mediator, inverse odds ratio weights were derived for each participant by taking the inverse of the predicted diet group – mediator OR. Regular meat-eaters (reference group) were assigned a weight of 1, and vegetarians were assigned the inverse odds ratio weights. Multivariable Cox regression models with weights applied were then fit to estimate the direct effect. The indirect effect was then calculated as the HR for the total effect divided by the HR for the direct effect. Percentage mediation (the proportion of the exposure – outcome association mediated by the mediator of interest) was calculated as [[ln(HR total) - ln HR direct] / ln(HR total)] x 100. 95% CI’s for total, direct, and indirect effects, as well as percentage mediation estimates, were estimated by bootstrapping their respective HRs using 300 replications. In line with existing recommendations, we report percentile-based CI’s (29, 30). Participants not in the binary diet group of interest (occasional meat-eaters and pescatarians) or with missing data for a variable required in each mediation analysis were excluded from that analysis.

# **Supplementary results**

## Diet group at recruitment and follow-up

The agreement of diet group in 57,730 participants with measures at recruitment and at least one instance of follow-up (using the latest available instance per participant) was generally high (Additional file 1: Table S4). Of 35,801 regular meat-eaters at recruitment, 27,277 (762%) remained regular meat-eaters, with 8,043 (22.5%) becoming occasional meat-eaters. Of 19,009 occasional meat-eaters at recruitment, 11,487 (60.4%) remained occasional meat-eaters, and 6,966 (36.6%) became regular meat-eaters. Of 1621 pescatarians at recruitment, 1306 (80.6%) remained pescatarian, with 157 (9.7%) becoming occasional meat-eaters, and 105 (6.5%) becoming vegetarian. Of 1223 vegetarians at recruitment, 1058 (86.5%) remained vegetarian, with 81 (6.6%) becoming pescatarian, and 50 (4.1%) becoming vegan. Of 76 vegans at recruitment, 54 (71.1%) remained vegan, with 19 becoming vegetarian (25.0%). The proportion of regular meat-eaters decreased over follow-up (35,801 (62.0%) to 34,284 (59.4%)), whilst the proportion of all other diet groups increased (Additional file 1: Table S4).

## Dietary characteristics at recruitment

Dietary intake of foods, beverages, and nutrients across diet groups at recruitment are summarised in Additional file 1: Table S5. Compared to regular meat-eaters, vegetarians and pescatarians ate more fruit and vegetables, wholegrains, and meat substitutes per day, and consumed fewer sugar-sweetened beverages. Consumption of dairy products was broadly similar across diet groups, though compared to regular meat-eaters, vegetarians consumed less milk (161.7 ml/day vs 108.9 ml/day) and more cheese (126.8 g/day vs 16.6 g/day).

Total protein intake was lowest in vegetarians (63.1 g/day) and highest in regular meat-eaters in both absolute terms (63.1 vs 84.8 g/day) and relative to body weight (0.91 vs 1.1 g/kg body weight/day). Vegetarians were also less likely to meet the recommended daily protein intake of 0.75 g/kg body weight/day, with 31.8% of vegetarians below this threshold compared to 14.8% for regular meat-eaters. Dietary calcium intakes were similar across diet groups, and on average, all groups exceeded the UK recommended intake of 700 mg/day (31). Unsurprisingly, vegetarians did not consume haem iron. Dietary iodine, niacin, selenium, vitamin B12, and vitamin D intakes were lower in vegetarians compared to other diet groups (iodine: vegetarians 161.0 µg/day vs average across all diet groups 221.3 µg/day; niacin: 29.3 mg/day vs average across all diet groups 38.3 mg/day; selenium: 34.8 µg/day vs average across diet groups 52.5 µg/day; vitamin B12: 3.7 µg/day vs average across diet groups 6.2 µg/day; vitamin D: 2.1 µg/day vs average across diet groups 3.6 µg/day). Dietary retinol intakes were also lower in pescatarians (345.8 µg/day) and vegetarians (338.9 µg/day) than in regular meat-eaters (498.1 µg/day).

## Descriptive characteristics at recruitment with varying restrictions

Characteristics of participants across diet groups at recruitment including or restricting to those with missing covariate data are shown in Additional file 1: Table S7. Compared to participants who were included in the study (with complete covariate data), participants with missing covariate data included a greater proportion of females, were less likely to have a degree, had a lower Townsend Deprivation Index, had lower values for height, and included a greater proportion of postmenopausal women, despite small differences in age, across all diet groups. Vegetarians with missing covariate data included a higher proportion of Asian participants, reported higher physical activity levels, were more likely to report never having smoked, had a slightly higher BMI, and were less likely to have children than vegetarians with complete covariate data.
